# Enhanced Electrochemical Capacity of Spherical Co‐Free Li_1.2_Mn_0.6_Ni_0.2_O_2_ Particles after a Water and Acid Treatment and its Influence on the Initial Gas Evolution Behavior

**DOI:** 10.1002/cssc.202201061

**Published:** 2022-09-08

**Authors:** Florian Klein, Joachim Bansmann, Zenonas Jusys, Claudia Pfeifer, Philipp Scheitenberger, Manuel Mundszinger, Dorin Geiger, Johannes Biskupek, Ute Kaiser, R. Jürgen Behm, Mika Lindén, Margret Wohlfahrt‐Mehrens, Peter Axmann

**Affiliations:** ^1^ Zentrum für Sonnenenergie- und Wasserstoffforschung Baden-Württemberg (ZSW) Helmholtzstrasse 8 D-89081 Ulm Germany; ^2^ Institute of Surface Chemistry and Catalysis Ulm University Albert-Einstein-Allee 47 D-89081 Ulm Germany; ^3^ Institute of Theoretical Chemistry Ulm University Albert-Einstein-Allee 11 D-89081 Ulm Germany; ^4^ Institute for Inorganic Chemistry II Ulm University Albert-Einstein-Allee 11 D-89081 Ulm Germany; ^5^ Electron Microscopy Group of Materials Science Ulm University Albert-Einstein-Allee 11 D-89081 Ulm Germany; ^6^ Helmholtz Institute Ulm Electrochemical Energy Storage (HIU) Helmholtzstraße 11 D-89081 Ulm Germany

**Keywords:** acid treatment, DEMS measurements, Li-rich cathode materials, lithium-ion batteries, surface modification

## Abstract

Li‐rich layered oxides (LRLO) with specific energies beyond 900 Wh kg^−1^ are one promising class of high‐energy cathode materials. Their high Mn‐content allows reducing both costs and the environmental footprint. In this work, Co‐free Li_1.2_Mn_0.6_Ni_0.2_O_2_ was investigated. A simple water and acid treatment step followed by a thermal treatment was applied to the LRLO to reduce surface impurities and to establish an artificial cathode electrolyte interface. Samples treated at 300 °C show an improved cycling behavior with specific first cycle capacities of up to 272 mAh g^−1^, whereas powders treated at 900 °C were electrochemically deactivated due to major structural changes of the active compounds. Surface sensitive analytical methods were used to characterize the structural and chemical changes compared to the bulk material. Online DEMS measurements were conducted to get a deeper understanding of the effect of the treatment strategy on O_2_ and CO_2_ evolution during electrochemical cycling.

## Graphical abstract

**Surface treatment**: Co‐free Li_1.2_Mn_0.6_Ni_0.2_O_2_ cathode material was treated with acid or water to modify the surface. A subsequent calcination step at lower temperature provided materials with high capacity (up to 270 mAh g^‐1^) and cycling stability, whereas high temperatures lead to deactivation.

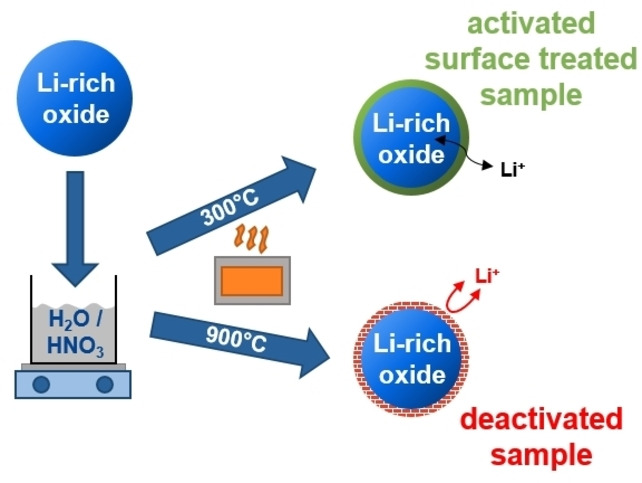

## Introduction

Driven by the climate change, the importance of energy storage increased in the last years due to the expansion of renewable energies and the need for balancing their power fluctuations.^[1]^ Additionally, different sectors, for example, the automotive industry, undergo a deep structural change from combustion engines to electric drive. To fulfill the requirements of the different sectors, lithium ion batteries (LIBs) are one of the most promising candidates in various applications due to their long cycling life, high energy density and strongly decreasing costs over the last years.^[2]^ A further increase of the energy density can be achieved, for example, with the development of new cathode materials like Li‐rich layered oxides (LRLO), which may increase the discharge capacity and the voltage window compared to commercially available cathode materials like LiCoO_2_ (LCO) or LiNi_0.8_Mn_0.1_Co_0.1_O_2_ (NCM811). Simultaneously, the amount of critical raw materials like Co or in the future Ni can be reduced to lower both raw material costs and the environmental footprint.

In general, LRLOs with the chemical formula of *x* Li_2_MnO_3_ ⋅ (1−*x*) LiMn_1−*y−z*_Ni_*y*_Co_*z*_O_2_ have to be taken into consideration as next generation cathode materials. Their high discharge capacity (over 250 mAh g^−1^) with an average discharge potential of >3.5 V vs. Li/Li^+^ is attributed to the combination of cationic and anionic redox reactions.[^3^, ^4^] The detailed mechanism of the anionic redox reaction and the involved active species of oxygen is still under debate.[^5^, ^6^] Furthermore, the structure of LRLOs is also under discussion. In 2005 Thackeray et al. suggested that the LRLO is a two component nanocomposite material consisting of monoclinic Li_2_MnO_3_ and a rhombohedral LiTMO_2_.^[7]^ Due to the structural relationship of the two phases, the monoclinic lattice can be described as a rhombohedral one (phase prototype: α‐NaFeO_2_) with a honeycomb superstructure. As a consequence, the material can be additionally classified as a solid solution with the general formula of Li_1.2_Mn_0.8−*y*−*z*_Ni_*y*_Co_*z*_O_2_.[^8^, ^9^]

However, the structure of the material strongly influences the redox‐mechanism. Most reported materials show a large irreversible capacity loss during the first cycle, a poor rate capability, a large hysteresis due to the anionic redox system and voltage fading over the cycle life.^[5]^ During the first delithiation, the Li_2_MnO_3_ part is electrochemically activated and the structure of the material changes into the electrochemically active Li_x_MnO_2_ phase. This goes along with the release of some lattice and surface oxygen.^[6]^ The release of surface oxygen leading to surface rearrangement from layered over spinel to the inactive rock‐salt structure (LS transition) has been suggested as the most likely reason for the observed voltage fading during cycling.[^5^, ^10^] To mitigate these problems, different approaches have been tested, such as doping, coating and different surface treatments. Doping is used mainly to stabilize the host framework (e. g., Al, Fe),^[11]^ or expand the Li layer spacing (e. g., Mg, Ca),^[12]^ whereas coatings protect the surface from the direct interaction with the electrolyte and thus suppress side reactions.^[13]^ Commonly used coatings are, for example, oxides,^[14]^ phosphates[^13^, ^14^, ^15^, ^16^] or fluorides.[^15^, ^17^] Another approach involves a surface modification during the synthesis route, in order to generate a thin spinel layer on the surface. The spinel phase increases the electrochemical performance due to a higher Li^+^ ion diffusion coefficient and the better electrical conductivity.[^18^, ^19^] Typical synthesis procedures include a chemical Li^+^ extraction, followed by a structural rearrangement step in the furnace, using different reactants: oxidizing or reducing agents like Na_2_S_2_O_8_,^[20]^ SO_2_,^[21]^ or N_2_H_4_ ^[22]^ as well as acids like HNO_3_,^[23]^ H_2_SO_4_,[^24^, ^25^] SO_3_,^[26]^ and organic acids,[^19^, ^27^, ^28^] or a simple water treatment.^[29]^

**Figure 1:**
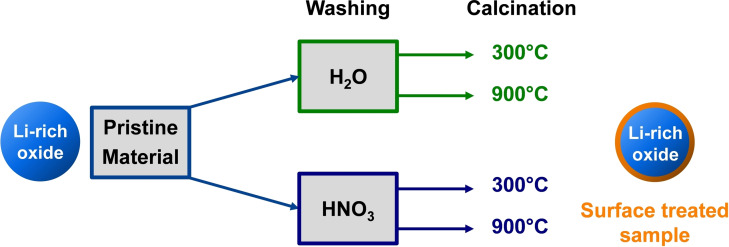
Schematic overview over the post treatment steps.

In this work, defined spherical precursor particles were synthesized in a continuous coprecipitation reaction, followed by an annealing step in the presence of Li^+^ (Figure 1). Inspired by the literature, the as synthesized Co‐free Li_1.2_Mn_0.6_Ni_0.2_O_2_ material was treated in a two‐in‐one procedure with water or HNO_3_, followed by a thermal treatment at 300 °C or 900 °C to largely remove surface impurities from the calcination step, and to simultaneously activate the material chemically. All process steps from precursor coprecipitation to the post‐treatment were selected with regard to the possibility of upscaling into the kg scale. The main objectives of this work are to map out and understand the relationship between the treatment procedure, in which the annealing temperature acts as a critical parameter, and the electrochemical cycling stability, the structural changes with respect to the primary and secondary particle architecture as well as the resulting gas evolution during the first cycles.

## Results and Discussion

The as‐prepared spherical, Co‐free material (labelled as LRLO) with the general formula of Li_1.2_Mn_0.6_Ni_0.2_O_2_ was used as a reference material. The treated samples were named according to their treatment steps and calcination temperatures. Samples denoted W300 and W900 were washed with water, whereas A300 and A900 were treated with acid, respectively. Adding the LRLO powder into water instantaneously led to the formation of an alkaline suspension with a pH≈11.6, reflecting the dissolution of Li^+^ and other surface species into the washing solution (Figure S1). The addition of HNO_3_ to the aqueous suspension led to an enduring decrease of the pH≈6–7, accompanied by the formation of small gas bubbles, due to the formation of CO_2_ from CO_3_^2−^ species. A pH curve measured during the addition of the powder to an acidic solution with the same amount of HNO_3_, added slowly during the post‐treatment step, is presented in the Supporting Information (Figure S1). Possible Li^+^ sources are water‐soluble surface impurities and the exchange of Li^+^ ions with H^+^ in the crystal structure of LRLO. Surface impurities like hydroxides and carbonates were quantified by acid‐base titration (Figure S2). The results verify that both washing procedures led to a pronounced decrease in the amount of carbonate and hydroxide species.

To quantify the metal leaching during the washing steps, inductively coupled plasma optical emission spectroscopy (ICP‐OES) was used (Table 1). The amount of extracted Li^+^ ions per formula unit increases with increasing acidity of the washing media, whereas the loss of Li^+^ is very low using water. Water washing probably dissolves mainly surface species, while the acidic treatment extracts significantly more Li^+^ out of the crystal lattice. Additionally, the Ni/Mn ratio is also decreasing for the acid treated samples. This leads to the assumption that the acid leaches not only Li^+^ ions out of the structure, but also attacks the lattice of the host framework, which is implied by the significantly increased Ni^2+^ content in the washing solution (Table 2). Interestingly, the Mn ion leaching is negligible. The observed pH‐dependent dissolution behavior is comparable to the results reported by Dahn's group for Ni‐rich Li‐TM‐layered oxide materials, where a significant TM leaching was observed below a pH of about 8, and where the leaching increased with decreasing pH.^[30]^ Besides the main elements Li^+^, Ni and Mn ions, small amounts of Na^+^ impurities were found in the pristine material, which are residues of the coprecipitation process. The Na^+^ ions were likely incorporated into the grain structure of the carbonate precursor and cannot be removed even upon extensive washing. However, after lithiation and calcination the Na^+^ ions can be quantitatively washed out easily with the used post‐treatment steps. This suggests that most of the Na^+^ impurities had segregated out of the particle architecture and deposited on the surface during the synthesis process of LRLO.

**Table 1:** Results of elemental analysis by ICP‐OES given as molar ratios (Li+Mn+Ni=2), mean oxidation states (OS) of the transition metals (TM=Ni+Mn), the calculated oxygen stoichiometry and the experimental lattice parameters of the powders.

	Li	Mn	Ni	Na/TM	Ni/TM	Mean OS	Li_*x*_TM_2−*x*_O_*y*_ *y*	Lattice parameter	
								*a* [Å]	*c* [Å]
LRLO	1.22	0.59	0.19	0.08	0.246	3.59	2.01	2.858	14.257
W300	1.21	0.60	0.19	0.00	0.245	3.55	2.00	2.859	14.258
W900	1.21	0.60	0.19	0.00	0.246	3.53	2.00	2.860	14.262
A300	1.17	0.63	0.20	0.00	0.239	3.55	2.06	2.863	14.274
A900	1.16	0.64	0.20	0.00	0.240	3.50	2.05	2.849	14.264

**Table 2:** Quantitative ICP analysis of the washing solutions after the aqueous or acidic treatment of LRLO. The results were normalized to the dry mass of the washed powders.

	Li [‰]	Na [‰]	Ni [‰]	Mn [‰]
Aqueous treatment	0.91	13.7	<0.00008	0.00374
Acidic treatment	3.70	13.8	0.429	0.00050

**Figure 2:**
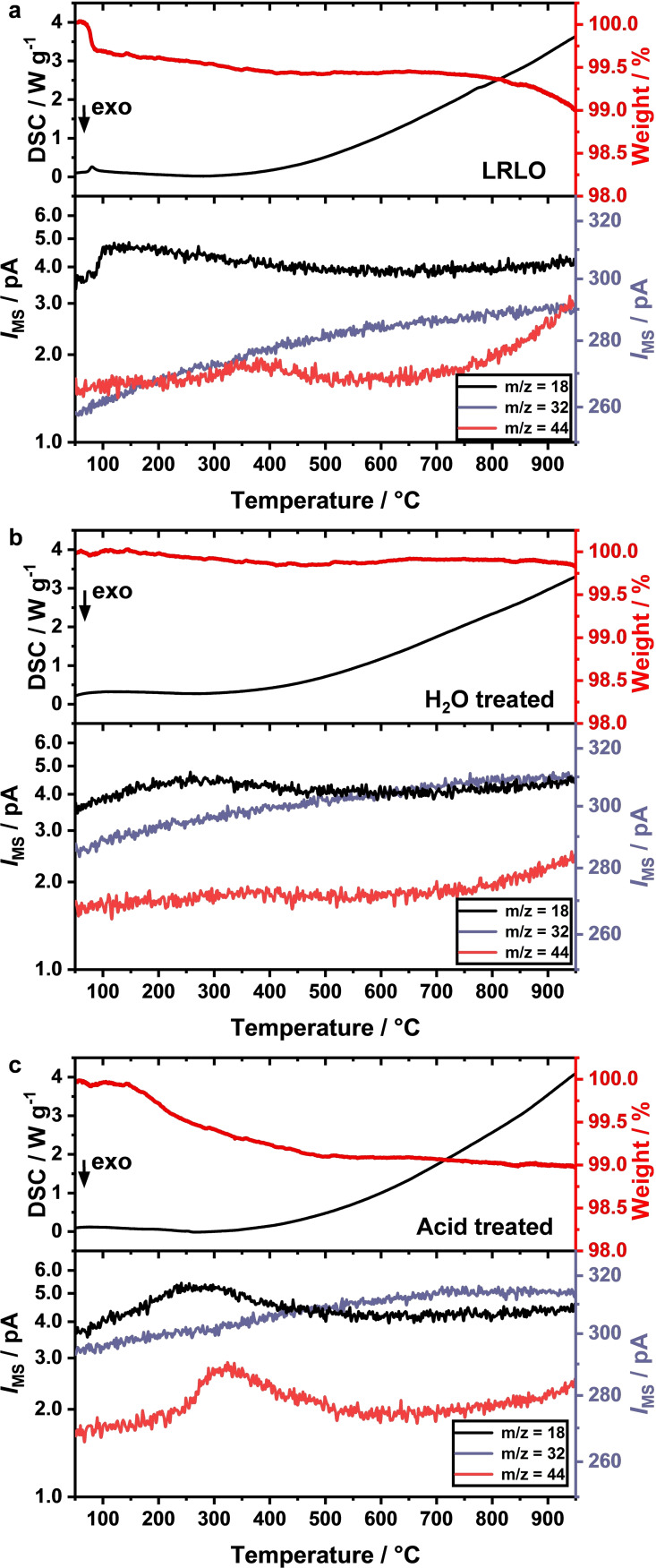
TGA‐DSC‐MS measured with a heating rate of 10 K min^−1^ under air for as‐synthesized LRLO (a), LRLO after aqueous washing (b) and LRLO after acidic treatment (c). The relative mass loss curves are shown in red and the DSC curves in black. The corresponding ion currents of the mass spectrometer are shown for m/z=18 (H_2_O, black), m/z=32 (O_2_, purple) and m/z=44 (CO_2_, red).

The second part of the post‐treatment, the calcination procedure after the washing step, was investigated using thermogravimetric analysis coupled with a mass spectrometer (TGA‐MS, Figure 2). The TGA curve of the pristine LRLO material (Figure 2a) is dominated by an initial mass loss of 0.5 % with a subsequent slight decrease in mass up to 400–500 °C, followed by a plateau. An additional mass loss was observed at temperatures exceeding 700 °C. The low‐temperature mass loss corresponds to the loss of crystal water from the Na_2_CO_3_ monohydrate, as illustrated by the increase of the H_2_O signal in the MS and the small endothermic peak in the differential scanning calorimetry (DSC) curve.^[31]^ Above 700 °C, decomposition of Li_2_CO_3_ followed by Na_2_CO_3_ occurs, as seen in the increasing CO_2_ signal.^[32]^ The broad CO_2_ peak in the temperature range of 300 to 500 °C, accompanied by a slow decrease of the H_2_O signal probably corresponds to the decomposition of basic Ni carbonate traces on the surface.[^33^, ^34^, ^35^] The O_2_ signal is dominated by the background signal of the air atmosphere. The monotone increase correlates with the increase of the N_2_ signal (*m/z*=28, Figure S3) and corresponds to a measurement artefact of the TGA‐MS setup. Unfortunately, the expected loss of O_2_ during the TGA experiment caused by the decomposition of possible protonated species cannot be observed nor excluded due to the already high MS signal from the oxygen containing air. Both washing treatments led to a pronounced change in the mass loss curve. The mass loss related to carbonate decomposition is mainly absent, which confirms the virtually quantitative elimination of Na_2_CO_3_ and Li_2_CO_3_. Only a very small amount of surface carbonates is still observed, indicated by the increasing CO_2_ signal above 700 °C. Possible sources are minor residues from an incomplete washing procedure or the formation of new carbonates upon contact with air.

The water washed material shows only a very small mass loss over the whole temperature range (Figure 2b). This we assign to the decomposition of Li_1.2‐*x*_H_*x*_Mn_0.6_Ni_0.2_O_2_ species, indicating some Li^+^/H^+^ exchange during the washing procedure.^[30]^

For the acid treated sample (Figure 2c), the corresponding mass loss is more pronounced (150–500 °C, ≈0.85 %). In combination with the increased H_2_O and CO_2_ signals, the decomposition of basic Ni carbonates to NiO is very likely.[^33^, ^34^, ^35^] With respect to the ICP results, this might be an indication that the acid treatment strongly attacks the LRLO lattice. Mainly Li^+^ and Ni^2+^ ions were dissolved, but most of the Ni^2+^ ions were likely deposited outside the LRLO crystal lattice as (oxo‐)hydroxides or form similar surface groups, which further react with air to basic Ni carbonates. Based on the TGA‐MS results and the corresponding literature,[^33^, ^34^, ^35^, ^36^] we suggest that the Ni surface species are fully decomposed at the calcination temperature of 900 °C to a rock‐salt like structure, whereas at 300 °C the surface species are only partially decomposed.

**Figure 3:**
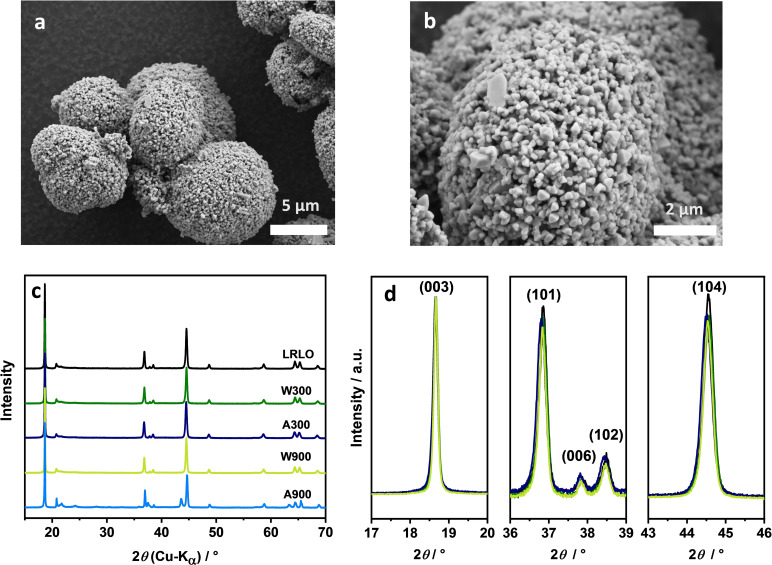
SEM images of LRLO (a, b). Powder XRD pattern of all the samples (c). XRD diffraction detail plots (d) of LRLO (black), W300 (green), A300 (dark blue) and W900 (light green).

Therefore, the structural and morphological changes after both treatment steps were further investigated, using scanning electron microscopy (SEM), powder X‐ray diffraction (XRD) and cerimetry (Figure 3). The SEM images (Figure 3a,b) of the pristine particles show a spherical morphology, derived from the spherical shape of the precursor. The secondary particles are agglomerates of primary particles with a few hundred‐nanometer size. Regarding the post‐treatment steps, there is no significant morphological difference (see Supporting Information Figure S4). The XRD diffraction patterns of all studied materials apart from the A900 material were similar, which is why they are discussed together in the following. All reflections can be assigned to the hexagonal crystal structure of the *α*‐NaFeO_2_ type with the space group *R‐3m* and the monoclinic Li_2_MnO_3_ type (*C/2m*) with indications for the presence of a Li_2_MnO_3_ type super‐structure, indicated by additional reflections in the 2*θ* range of 20–25° (Figure 3c).^[37]^ The layered structure is well defined, indicated by the splitting of (006) and (102) as well as (108) and (110) reflections.[^38^, ^39^] The (003), (101) and (104) reflections do not show shoulders or additional signals in their neighborhood indicating that the spinel phase content must be low (Figure 3d).[^38^, ^40^] TOPAS V6 was used for Rietveld refinement of the XRD data, using the Li_1.2_Mn_0.6_Ni_0.2_O_2_ structural model of Fell et al.^[41]^ Detailed results of the refinement and the corresponding plots are presented in the Supporting Information (Table S1, Figure S5). The lattice parameters *a* are in a comparable range *a*=2.858– 2.863 Å with the exception of A900 (*a*=2.849 Å), being significantly lower (Table 1). In contrast, the value of the *c* parameter is strongly dependent on the washing conditions. The largest difference is observed between A300 (*c*=14.274 Å), LRLO (*c*=14.257 Å) and W300 (*c*=14.258 Å). A possible explanation or the slight increase in the *a* parameter values after the treatments can be the adjustment to a new oxygen‐TM stoichiometry during calcination, due to the release of thermodynamically unstable oxygen from the crystal lattice to compensate a local loss of Li^+^ during washing, for example due to reduction of Mn^IV^ to Mn^III^. The general phenomenon is called reduction expansion.[^42^, ^43^] As a consequence, the cell volume increases and the activation energy for Li^+^ ion transport is likely decreased.^[44]^ However, the changes in the structure have to be very local, for example, on the surface of the primary particles, because the XRD patterns (bulk method) show that the hexagonal structure remains intact without visible structural changes.

The diffraction pattern of A900 differs strongly from that of the other powders. Additional reflections appear, which can be assigned to the rock salt structure of the type Mg_6_MnO_8_ (225) with a chemical composition of most probably Ni_6_MnO_8_.[^45^, ^46^] Additionally, the reflections originating from a Li_2_MnO_3_‐type super‐structure between 20 and 25° 2*θ* are well defined and have a higher intensity as compared to the other materials studied. Compared to A300, both lattice parameters decrease dramatically. In combination, this implies that some phase transition or phase separation, respectively, occurred during the high‐temperature calcination step. A more detailed discussion about A900 will follow in a separate section.

To verify the reduction expansion of the lattice parameters, TM mean oxidation states (OS) were investigated by cerimetric redox back titration. Generally, the TM mean OS decreases with the treatment steps compared to LRLO (Table 1). For an easier comparison of the results, the values were converted into the ratio of oxygen vs. metal with respect to the formula unit of the layered oxide Li_2−*x*_TM_*x*_O_*y*_ (*y*=2.00), using the Li/TM ratio obtained from ICP. Additionally, theoretical values for LiTM_2_O_4_ (*y*=2.67) and Ni_6_MnO_8_ (*y*=2.29) were calculated. The value of LRLO *y*=2.01 corresponds to the theoretical one of *y*=2.00, which fits well to the expectations. None of the water‐treated samples show stoichiometric differences as compared to pristine LRLO. In contrast, the calculated amount of oxygen per formula unit of the acid treated samples A300 and A900 increased slightly. This indicates the presence of an O‐richer crystallographic phase, like spinel‐type (A300) or Ni_6_MnO_8_ (A900), in addition to the layered phase, which is in good agreement with the XRD results.

**Figure 4:**
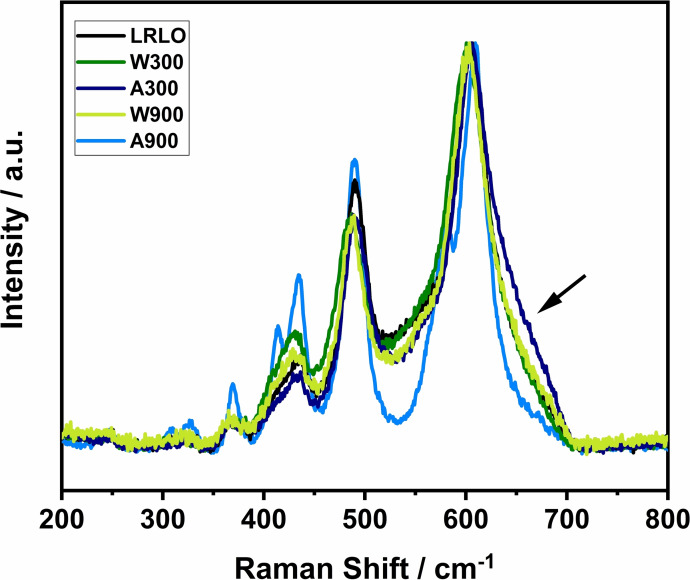
Raman spectra of the investigated powders (λ=532 nm, P=1.5 mW). The spectra were normalized to the vibrational band at approximately 600 cm^−1^.

Deeper insight can be gained by more surface sensitive and high‐resolution methods like X‐ray photoelectron spectroscopy (XPS) as well as Raman and transmission electron microscopy (TEM). In contrast to XRD, Raman microscopy is sensitive to short range order and allows to differentiate between different coordination geometries and oxidation states.^[47]^ If the LRLO structure is a nanocomposite of LiTMO_2_ and Li_2_MnO_3_, the Raman spectrum should show lattice vibrations of both structures. The LiTMO_2_ compound with the space group *R‐3m* has two vibrational modes: the in‐plane O−M−O bend vibrations E_g_, where the oxygen ions vibrate in parallel to the Li/TM layers and the out‐of‐plane O−M stretching vibration mode A_1g_, where the oxygen ions vibrate in opposite directions along the *c*‐axis.[^47^, ^48^] In case of a solid solution of different metals, for example LiNi_1‐x_Mn_x_O_2_, each type of TM ion bond has separate E_g_ and A_1g_ vibrations. Typical values for NCMs are around 500 cm^−1^ (E_g_) and 600 cm^−1^ (A_1g_), depending on the ratio of the TMs.^[49]^ The symmetry of Li_2_MnO_3_ is lower and therefore more vibration bands can be excited at 612 (A_g_), 568, 493, 438, 413, 369, 332, 308 and 248 cm^−1^.^[50]^ The measured spectrum of pristine LRLO shows a superposition of all modes (Figure 4), which is therefore in accordance with the literature. It is important to note that the vibration bands of Li_2_MnO_3_ between 200 and 440 cm^−1^ are less pronounced in LRLOs compared to the phase pure compound due to local structural differences like the exchange of Mn with Ni or Li/Ni disordering. Furthermore, this is a possible reason for band broadening and a shift to lower wavenumbers (red‐shift).^[9]^ Figure 4 shows a selected spectral range to focus on the important M−O vibrations. Additional vibrations beyond 800 cm^−1^ are detected only for LRLO at approximately 1080 cm^−1^ (compare Figure S6). This high wave‐number band can be attributed to the symmetrical stretching vibrations of the carbonate ions in Na_2_CO_3_.^[51]^ It suggests that remaining sodium from the precipitation process is present as a separate, water‐soluble phase and therefore can be removed completely during the post treatment steps, which confirms the ICP‐OES results. The M−O vibrations supports the different observations of the bulk characterization. The spectra of LRLO, W300 and W900 show no significant differences between each other. In the spectrum of A300, an additional shoulder at ≈650 cm^‐1^, marked with a black arrow, can be observed. It shows the presence of some shorter Mn‐O or Ni‐O bonds in the structure, most likely due to the formation of some spinel domains in the crystal lattice during the Li^+^ leaching and the low temperature treatment step.[^9^, ^50^, ^52^] Further vibration bands assignable to spinel structures are not visible, because they have lower intensity and are hidden under the signals of the main phase.^[50]^

**Figure 5:**
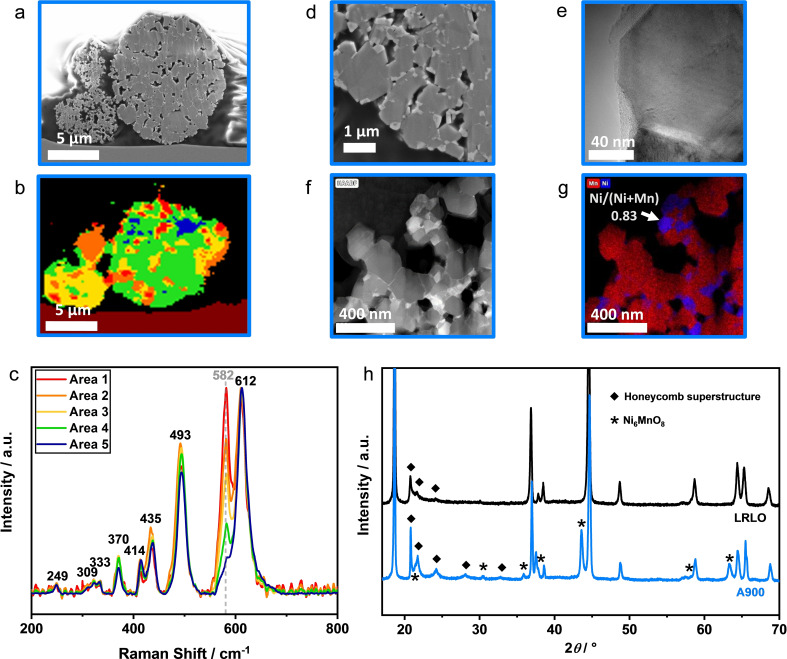
Detailed investigation of A900: SEM image of particle cross‐section (a), Raman mapping of the same particle cross‐section, clustering areas with similar spectra (b) and corresponding Raman spectra (c), SEM image (d) with higher magnification of (a), HRTEM image of a crystallite (e), HAADF STEM image (f) with corresponding elemental distribution of Mn and Ni determined with EDX (g), detailed XRD analysis (h) with phase identification of Ni_6_MnO_8_ (marked with *, reference: Taguchi et al.^[46]^).

Fundamentally different is the Raman spectrum of A900. The vibration bands are sharper and well‐defined, especially in the range of 300–450 cm^−1^. The most intense band is slightly shifted to higher wavenumbers compared to the other samples. Furthermore, an additional signal at 582 cm^−1^ can be observed. A cross‐section of the material was prepared and studied by a combination of SEM and Raman microscopy (Figure 5a–c), correlating structural information (Raman) with higher resolution images (SEM). Similar Raman spectra were clustered into groups and presented in the mapping as areas with the same color (Figure 5b). The corresponding average Raman spectra (Figure 5c) show strong local differences in the signal intensity ratio between the vibration band centered at ≈582 cm^−1^ and the band at 612 cm^−1^, while the intensity ratios of the vibrational bands at 612, 496, 435, 414, 370, 333, 309 and 249 cm^‐1^ are approximately constant. This strongly indicates a mixture of two phases. The latter vibration bands can be assigned to the Li_2_MnO_3_ structure.^[50]^ Therefore, a single vibration mode for the second phase at ≈582 cm^−1^ remains. This is in good agreement with the literature values for Ni_6_MnO_8_, showing a single vibration mode between 580 and 585 cm^‐1^.^[53]^ With the additional reflections in the XRD (Figure 5f) and the strong decrease of the lattice parameters from A300 to A900, a phase separation into Ni_6_MnO_8_ and a Ni‐poor LRLO with a high Li_2_MnO_3_ content for A900 is very likely. The TM separation is already visible in the contrast of the SEM image as well as in the high‐angle annular dark field (HAADF) scanning (S)TEM images (Figure 5a,d). The Ni‐rich regions are brighter due to the higher atomic number *Z*, scattering more electrons.^[54]^ The Ni‐rich particles can be found on the surface of the primary particles and at the grain boundaries, which is clearly indicated additionally by the Raman mapping (high intensity of the vibration band at ≈582 cm^‐1^ in the bright areas of the SEM images). Energy dispersive X‐ray (EDX) mapping of the same TEM lamella supports these observations (Figure 5e). There are some local Ni enrichments, for example, with a Ni/TM ratio=0.83 and the predominant part of dark particles with Ni/TM ≈0.07. Taken the results together, A900 indicates that the material was strongly attacked by the acid, and that the leaching process destabilized the structure in a manner that the subsequently high‐temperature treatment, where especially the TM ions have a high mobility and move to the thermodynamically most stable lattice site, leads to phase separation. For W900, a similar reaction behavior is assumed to take place, but to a lower extent.

**Figure 6:**
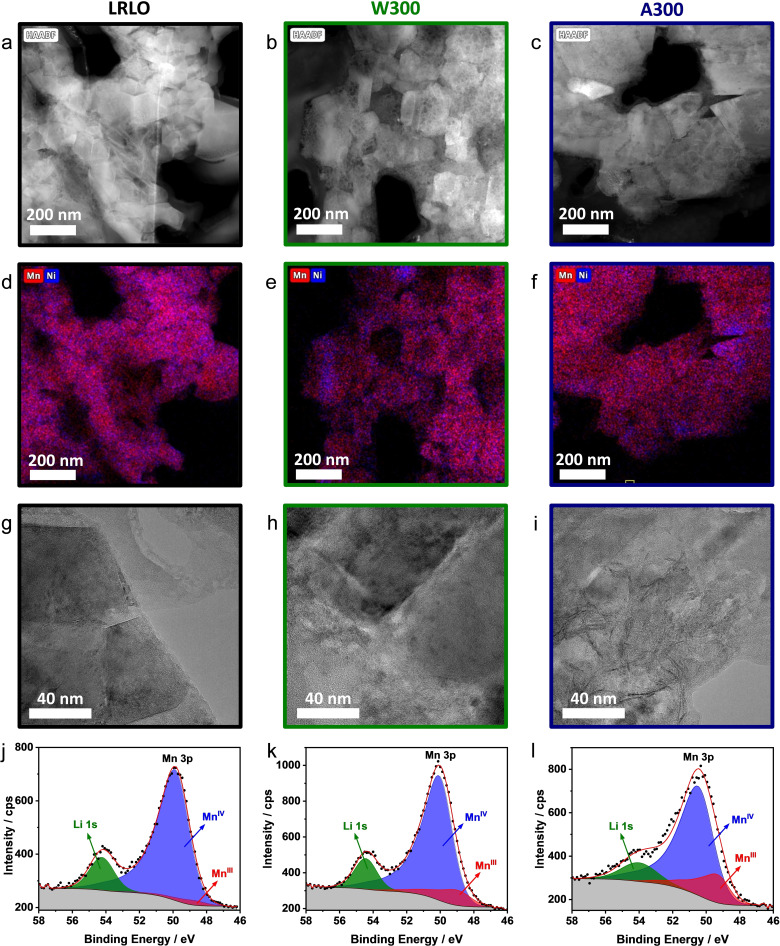
Detailed structural investigation of LRLO (black), W300 (green) and A300 (blue) with TEM (a–i) and XPS (j–l). HAADF STEM images (a–c), corresponding EDX elemental distribution of Mn and Ni (d–f), HRTEM images (g–i) and XPS spectra of Li 1s and Mn 3p (j–l).

This behavior helps to interpret the changes in the 300 °C treated samples, which will be discussed in the following part. The results of the TEM and XPS investigation of LRLO, W300 and A300 are presented in Figure 6. TEM lamellas were cut using a focused Ga‐ion beam (FIB). Typical and representative particles with diameters of around 15 μm were selected. The cuts were set in a way that the surface and the first few micrometers deep into the particles could be analyzed. The HAADF STEM images (Figure 6a–c) show the differences between the samples directly. LRLO consists of defined crystallites in the range of approximately 100–200 nm with a homogeneous surface and brightness. In contrast, the crystallite surface of W300 shows a large number of cavities and is generally inhomogeneous. The cavities originate most likely from the leaching process during the post‐treatment and the associated surface corrosion. A300 has a smoother surface, in which the single crystallites are difficult to identify. Additionally, the parts of the crystallites located directly adjacent to the surface or pores appear brighter compared to parts farther away. EDX results are consistent with these observations. After both acid and water treatment, a local Mn‐ and Ni‐enrichment can be observed whereas the pristine LRLO material has a homogeneous Mn−Ni distribution. High resolution (HR) TEM images (Figure 6g–i) show that the sharp edges of the well‐defined crystallites (LRLO) become smoother for W300 and A300. Furthermore, some of the surface layers of A300 are likely exfoliated or have pronounced stacking faults, which might be a reason for the increased Li^+^ and Ni^II^ concentrations in the washing solution. In the exfoliated areas, the accessibility to the Li^+^ ions is strongly increased, supporting washing out. The TM layers are most likely preserved by the hardly soluble Mn^IV^ ions. However, the in general higher mobility of the Ni^II^ ions probably leads to the depletion of Ni in the TM layer and facilitates the local Mn‐ and Ni‐enrichment and densification of the surface. Consequently, the results of the material treatment are strongly dependent on the pH: the higher the proton concentration in the washing solution, the stronger the surface reconstruction after the heat treatment.

To underline the experimental results of Raman measurements and cerimetry, the formation of spinel domains during the post treatment steps was characterized by X‐ray photoelectron spectroscopy (XPS) (Figure 6j–l). The surface sensitive method was chosen in addition to the bulk sensitive cerimetric titration with the idea to investigate the reconstruction of the surface after Li^+^ leaching. The oxidation states of Mn were determined through deconvolution of the Mn 3p spectra, using the approach of Ilton et al.^[55]^ with five single peaks for each Mn OS (III/IV). The latter peaks were fitted with defined parameters (e. g. peak shape, width, relative binding energy and intensity) adapted from the literature values.^[55]^ All components of each OS are shown in one peak for each OS for an easier interpretation of the spectra. The overlapping Li 1s signal was fitted with a single peak. Obviously, the surface Mn^III^ content increases from LRLO (4 %) over W300 (12 %) to A300 (20 %), while the Mn^III^ content decreases slightly from A300 to A900 (Figure S7a). The Li 1s peaks are positioned in a comparable range of the BE of 54.2±0.2 eV, corresponding to Li−O‐bonds in LRLOs.^[56]^ Nevertheless, the peaks of Li 1s and Mn 3p in A300 are not well separated, like in W300, due to a broadening of the Li peak. This can be related to the lower BE in spinel type material, for example LiMn_2_O_4_ with a Li 1s BE=53.7 eV.^[57]^ Further spectra are presented in the Supporting Information (Figure S7). A behavior similar to that of the Mn 3p states is observed also for the Mn 2p spectra (Figure S7b). Furthermore, we also recorded Ni 2p spectra, which showed no significant differences between the different materials (Figure S7c). They are dominated by a Ni 2p_3/2_ peak at around 854.7 eV, which is characteristic for Ni^II^.^[58]^

**Figure 7:**
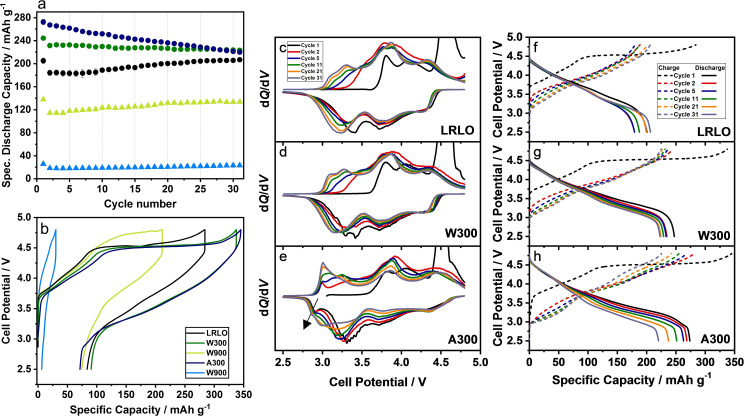
Specific discharge capacity (a) of C/10 cycling (first cycle C/20) and the galvanostatic potential curve (b) of the first cycle (C/20): LRLO (black), W300 (green), A300 (dark blue), W900 (light green) and A900 (blue). Corresponding dQ/dV plots (c–e) and potential curves of selected cycles (f–h): 1^st^ (black), 2^nd^ (red), 5^th^ (blue), 11^th^ (green), 21^st^ (yellow) and 31^st^ (grey).

After the detailed structural and chemical analysis of the powders, the influence of the chemical treatments on their electrochemical behavior was studied (Figure 7). The active materials were cycled against Li metal in a potential range between 2.5 and 4.8 V with C/10 (1 C=250 mA g^−1^). Due to the slow electrochemical activation step at approx. 4.5 V, the first cycle was conducted at a lower C‐rate of C/20. The specific discharge capacity of LRLO is 184 mAh g^−1^ in the second cycle (Figure 7a) and does not reach the values reported in the literature for this material class, for example 240 mAh g^−1^ (C/10, 2.0–4.8 V).^[59]^ Additionally, there is an increase of the capacity upon cycling, which indicates an activation of the cathode material. With respect to their electrochemical behavior, the treated samples can be separated into two groups. The materials with a post‐treatment calcination temperature of 300 °C (A300 and W300) reach higher values, whereas W900 and A900 were deactivated. Especially the deactivation of the latter material is very pronounced. From the antecedent characterization, this can be assigned to the structural reorganization during high‐temperature treatment. The lower electrochemical activity might be due to the presence of Ni‐rich rock‐salt structures, like Ni_6_MnO_8_, in the grain boundaries and on the surface of the primary particles. This layer can probably block the lithium diffusion pathways and deactivate the material electrochemically. Most likely, the same happens for W900, but to a lesser extent. A300 shows a very high specific discharge capacity of 267 mAh g^−1^ in the 2^nd^ cycle as compared to W300 (231 mAh g^−1^), but the capacity decreases rapidly during further cycling. W300 has a stable cycling behavior as well as LRLO. This is highlighted in more detail in the rate capability test and subsequent cycling at 1 C (100 cycles, Figure S8). A closer look at the voltage profiles and d*Q*/d*V* plots of LRLO, W300 and A300 helps to understand the different behavior of these materials. According to general knowledge, during the first delithiation Ni ions are oxidized in the sloping area of the voltage profile.^[6]^ At approx. 4.5 V a plateau is reached, where the Li_2_MnO_3_ domains are activated. Lithium and oxygen species are extracted, which provides further capacity in addition to the TM redox system and the TM metals are rearranged.^[60]^ During the subsequent re‐lithiation process Li^+^ mainly re‐occupies positions in the Li^+^ layer.[^5^, ^61^] LRLO and W300 have the typical charge and discharge potential characteristic of the Li‐rich material during the first cycle (Figure 7b). The voltage profiles only differ in length, but not fundamentally in the ratio of the sloping against the plateau region. If they were normalized on their maximum specific charge capacity, the 1^st^ cycle charging profile of LRLO is comparable to that of W300. This is in accordance with the d*Q*/d*V* plots of both materials (Figure 7c,d). A voltage shift towards higher or lower values cannot be observed there, but a closer look shows that the intensity of the oxidation peak beyond 4.2 V increases during the following cycles for LRLO, whereas for W300 it decreases. Secondly, the d*Q*/d*V* plot of LRLO shows an additional signal at the end of charge during the first few cycles. The combination of the slight capacity increase during cycling and the information from the d*Q*/d*V* plots indicates that only a part of LRLO material participates in the charge and discharge processes. Clogged diffusion paths in the secondary particle due to residues of the synthesis (e. g. Na_2_CO_3_ or Li^+^ species) or in the primary particles may hinder electrochemical access to the other part of the material. During further cycling, some species can react (e. g., in the d*Q*/d*V* signal at the end of charge) and form additional diffusion pathways, which makes further active material accessible.

The first cycle potential curves of A300 are similar to those of the previous samples with one difference in the discharge profile, which has additional capacity in the region of *E*≈2.7–3.1 V and in the following cycles (Figure 7h). In the corresponding d*Q*/d*V* plot (Figure 7e), a peak in this potential range (black arrow) is observable, which increases in intensity during further cycling. During delithiation the peak between *E*≈2.8–3.2 V increases, too. Both are assigned in the literature to the Mn^III^/Mn^IV^ redox couple of a spinel‐like phase,[^27^, ^62^, ^63^] which confirms the results already presented like, for example, a higher Mn^III^ content in the XPS spectra or the additional shoulder in the Raman signal. Furthermore, most of the other d*Q*/d*V* peaks differ as well. The oxidation peak at *E*≈4.5 V is slightly shifted to higher potential and the beginning of discharge starts earlier and smoother. Especially in the Ni redox active region above *E*≈3.2 V,^[63]^ the discharge peak intensity decreases strongly over cycling. The potential curves and the d*Q*/d*V* plots of the long‐term cycling experiments, which show a strongly increased capacity for A300 in the potential range between *E*≈2.8–3.2 V after 145 cycles (Figure S8c,d), underline this hypothesis. This can be interpreted as a faster aging process due to the rapid transformation from a layered into spinel‐like and rock‐salt structure. One reason for this phase transformation might be the local enrichment of Ni in the surface‐near areas, shown in the TEM analysis, and the Mn‐enrichment of the bulk with a higher amount of Li_2_MnO_3_ domains. The increase of Li_2_MnO_3_ domains is in accordance with the reported characteristics of the d*Q*/d*V* data of Zhang et al., varying the ratio between (*x*) Li_2_MnO_3_ and (1−*x*) LiMn_0.5_Ni_0.5_O_2_ (0.3≤x≤0.6).[^60^, ^64^] A second possibility is the structural instability of the surface‐near region after the acid treatment, which is indicated by the phase separation at 900 °C into rock‐salt and Li−Mn‐rich layered structures (A900). At 300 °C, a complete structural rearrangement is unlikely, but during subsequent cycling electrochemically induced oxygen release and repeated (de‐)lithiation are likely to further destabilize the structure, which leads to a strong surface densification and a decrease of the bulk accessibility.

**Figure 8:**
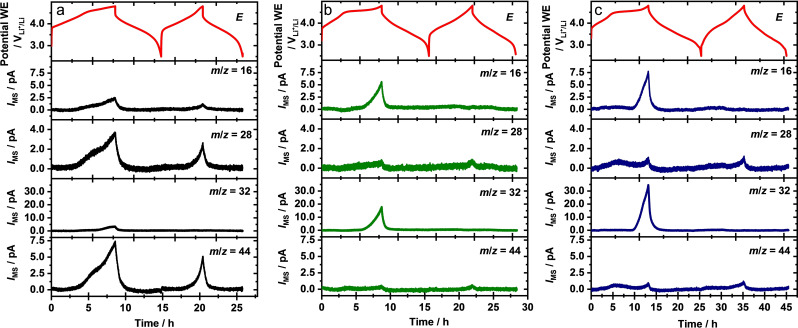
DEMS measurements of LRLO (a), W300 (b) and A300 (c) in half cell configuration (C/10) during the first two full cycles using a membrane inlet to transfer the gaseous products to the mass spectrometer: Cathode potential curve (upper panel) as well as mass spectrometer signals (remaining panels) for m/z=16, 28, 32 and 44 vs. time.

More information about possible gas evolution in the electrochemical activation of these materials and the influence of the different treatment steps thereon was obtained by DEMS measurements (Figure 8). They were performed under galvanostatic conditions (*I* ≈C/10), to be comparable with the electrochemical measurements in coin‐cells (for details see Experimental section). Most important, they were conducted under truly differential conditions, as the working electrode was directly deposited on the membrane inlet to the mass spectrometer chamber, which allows a fast transfer of the gaseous products to the mass spectrometer (time resolution of ∼2 s).^[65]^ Therefore, the signals can be directly correlated with the formation rates of the respective gases. To the best to our knowledge, such DEMS measurements are applied here for the first time. They differ by their much higher time resolution from the commonly used carrier gas assisted online mass spectrometry (OEMS) approach.[^4^, ^61^, ^66^, ^67^, ^68^]

The potential curves in the upper panel (Figure 8, red lines) are comparable to the electrochemical data obtained from coin cells that were shown before (Figure 7). The transients of selected ion currents presented in the other panels, which were acquired simultaneously, are plotted in the lower panels. They provide information on the gas formation rates from the active materials. Qualitatively, they clearly indicate the formation of O_2_ (*m*/*z*=32, with a fragment at *m*/*z*=16) and CO_2_ (*m*/*z*=44, with fragments at *m*/*z*=28 and *m*/*z*=16) in the range of high potentials.^[69]^ For none of the three powders we observed an O_2_ signal in the second cycle. The peak shapes and intensities differ, however, significantly for the different electrodes, with the amount of released O_2_ increasing in the order LRLO < W300 < A300. CO_2_ formation is also most pronounced in the first cycle, but occurs to a lower extent also in the second cycle. Comparable results had been reported by Streich et al. for Co‐containing LRLO/SFG6 full cells.^[70]^ In contrast to the O_2_ signal, the amount of CO_2_ formation is highest for the pristine LRLO, and much lower for the treated samples.

The much higher amount of O_2_ formation after pre‐treatment as compared to pristine LRLO seems to be in contrast to findings reported in the literature. Ramakrishnan et al. reported that acid washing and subsequent drying (T_max_=135 °C) results in complete suppression of O_2_ evolution during charging of Co‐containing LRLO material and an almost complete suppression of CO_2_ evolution.^[24]^ They explained the latter by a combination of partial removal of Li_2_CO_3_ (surface) impurities and surface passivation against electrolyte decomposition.^[24]^ Qiu et al. reported a strongly reduced O_2_ and CO_2_ evolution from a post‐treated Co‐containing LRLO material during charging, too.^[4]^ In their case, the electrode material reacted with CO_2_ to create O‐vacancies, followed by washing and drying to remove the Li_2_CO_3_ surface layer partly formed during this treatment. They attributed the suppression of gas evolution to the formation of O‐vacancies in the surface‐near region, which inhibit O_2_ evolution. This should also cause the inhibition of CO_2_ evolution, which according to these authors results from reaction between oxygen radicals and electrolyte.^[4]^ Because the results of the redox titration indicates the absence of O‐vacancies in the series of powders investigated here, it is assumed that during the thermal treatment under air at 300 °C and 900 °C, respectively, possible vacancies are removed and/or more thermodynamically favored phases are formed, for example, a spinel‐like phase. Consequently, the results of the DEMS measurements of LRLO, W300 and A300 are hardly comparable with the above studies.

The present result of a lower O_2_ evolution from the pristine LRLO sample as compared to the post‐treated W300 and A300 electrodes can be explained by a combination of less electrochemical oxygen removal and a higher conversion of the evolving reactive oxygen into several products. The first effect is supported by the observation of a lower capacity for the pristine sample, which we mainly attribute to a lower electrochemical accessibility of the surface regions due to the Na_2_CO_3_ deposits and a possible lower particle surface. The second contribution can result from a partial trapping of reactive oxygen species below or in the Na_2_CO_3_ impurities, provided that these species have a sufficiently long lifetime. This will not only reduce the measured O_2_ signal, but also allow a more efficient reaction between the evolved reactive oxygen and the electrolyte, in good agreement with the significantly more pronounced CO_2_ evolution on this sample as compared to the treated samples. The absence of measurable O_2_ evolution in the second cycle in all three samples tested here corresponds to previous findings for comparable systems,[^61^, ^67^, ^70^] where this was related to complete depletion of removable oxygen during activation. However, it cannot be excluded that there is some further oxygen release during subsequent cycling, which directly reacts with the electrolyte. Comparing with the d*Q*/dV signals of the electrochemical cycling above 4.5 V, this effect is likely more pronounced for LRLO and A300.

The increase of the O_2_ signal from W300 to A300 can be explained by the structural instability of the material before the calcination step. Nakamura et al.^[42]^ investigated the influence of vacancies on the stability of Li_1.2_Mn_0.6_Ni_0.2_O_2‐*δ*_ and reported a phase separation for *δ* >0.042 into Ni_6_MnO_8_ and a layered Ni‐deficient Li−Mn enriched Li(Li,Mn,Ni)O_2−*δ*_ phase after calcination above 400 °C under atmosphere with reduced oxygen partial pressure. In A900, a similar phase segregation was observed, leading to the assumption that the acid treatment leads probably to the formation of O vacancies. After the calcination step at 300 °C, A300 is most probably in a metastable state. During electrochemical cycling a phase transformation into a thermodynamically more favored equilibrium is supposed, which is accompanied by a strong oxygen loss in the structure. Furthermore, the TEM investigation of A300 has shown a formation of Ni‐enriched and Mn‐enriched domains, which leads to the assumption that the content of oxygen releasing Li_2_MnO_3_ domains is increased. Both explanations are in accordance with the faster electrochemical aging behavior of A300.

The significantly lower amount of CO_2_ evolution on the post‐treated samples as compared to pristine LRLO is explained by the essentially complete removal of Na_2_CO_3_ after washing. In that case, CO_2_ formation is only possible by reaction with the electrolyte, whereas for the pristine LRLO electrode CO_2_ formation is also possible by decomposition of Na_2_CO_3_, for example, by chemical reaction with H^+^ species.^[66]^ A more detailed analysis of the CO_2_ peak shapes and potential ranges is given below.

**Figure 9:**
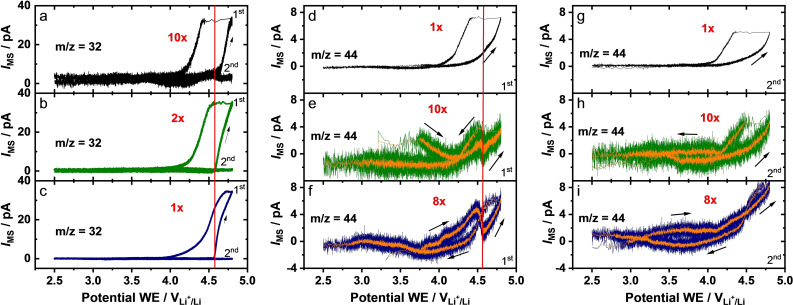
Mass spectrometric signals for m/z=32 and m/z=44 from Figure 7, replotted as a function of the potential for m/z=32 (a–c, 1^st^ and 2^nd^ cycle), m/z=44 (d–f, 1st cycle) and m/z=44 (g–i, 2nd cycle). The same material is arranged horizontally in the same line and in the corresponding color: LRLO (black, topmost panel), W300 (green, middle panel) and A300 (blue, bottom panel). Averaged curves (10 points average) have been added for better clarity over the signal course of some graphs in orange. The enlargement factors are given in red for better comparability.

More detailed information on the potential dependence of the gas evolution is obtained from Figure 9, where the main mass spectrometric signals (*m/z*=32 and *m/z*=44) are plotted as a function of the potential. For all three electrodes, O_2_ release starts at approximately ≈4.6 V, followed by a steep increase of the signal up to the upper potential limit of 4.8 V. Based on previous reports for similar materials,[^61^, ^68^, ^71^] it is assigned to the electrochemical activation of the Li_2_MnO_3_ domains present in the materials. Interestingly, the increase of the *m*/*z*=32 ion current vs. potential differs from the exponential behavior expected from the common Butler‐Volmer kinetics in a potentiodynamic scan at constant potential scan rate. The increase of the signal starts rapid upon 4.6 V, with a slight decrease of the slope at the end of charge. This is more pronounced for the treated samples. Switching from the end of charge to the beginning of discharge, the potential very rapidly (within few seconds) shifts to approximately 4.4 V at a nearly constant O_2_ formation rate for the LRLO sample. For the W300 and A300 electrodes, the potential shift is less pronounced, reaching approximately 4.5 and 4.7 V, respectively. The following strong signal decay deviates for all samples from an exponential decay with decreasing potential, changing to a more sigmoidal shape. The background level is reached at approximately 4.0 V. In the second cycle, the *m/z*=32 signal remains at the background level, irrespective of the applied potential. As it has been derived from a mass spectrometry study using ^18^O labeled layered Li[Li_0.2_Ni_0.2_Mn_0.6_]O_2_ material,^[72]^ it is assumed that the O_2_ originates mainly from the active electrode material rather than from the organic carbonate based electrolyte.

The differences in shape of the O_2_ evolution curves of LRLO, W300 and A300 are predominantly caused by differences in their potential curves and the corresponding potential variation rates. As evident from the potential curves (Figure 8), these differences are more pronounced for the W300 and A300 electrodes, in particular for the latter one. The strong increase of the O_2_ evolution starts in the flat potential plateau region with a low potential variation rate and ends up with a high potential variation rate at the end of charge, which explains the decreased slope of the O_2_ signal (Figure 9a–c). The differences in the potential drop at the beginning of discharge and in the subsequent signal decay of the oxygen can be explained by the comparison with the d*Q*/d*V* data (Figure 7c–e), since in contrast to LRLO, A300 shows a lower overpotential and smoother change potential variation rate.

Moving on to the CO_2_ signal, a pronounced signal for the LRLO electrode with an onset at approximately 4.1 V, well below the onset of O_2_ evolution (Figure 9d), is found in the first cycle. This is followed by an almost exponential increase above about 4.6 V. In contrast, the CO_2_ signal of A300 is significantly lower and clearly shows a first increase up to 4.4 V and a second one above 4.6 V, with a local minimum in between (Figure 9f). A similar, but less pronounced characteristic curve is observed for W300 (Figure 9e). It can be assumed that both maxima of W300 and A300 reflect contributions from different processes, as it had been proposed in previous studies.[^61^, ^68^] In those studies the authors suggested that the CO_2_ evolution at lower potentials is either caused by the oxidation/decomposition of (surface) impurities[^68^, ^73^, ^74^, ^75^, ^76^] or by oxidation of the electrolyte by high‐valence Ni ions.^[61]^ The results of an operando mass spectrometry study by Luo et al.,^[72]^ using ^18^O labeled layered Li_1.2_Ni_0.2_Mn_0.6_O_2_ material with Li_2_CO_3_ impurities, showed that the CO_2_ evolution observed for this material includes significant amounts of labelled C^18^O^16^O. They assumed that this originated from the oxidation of electrolyte with released lattice oxygen species, over the entire range of CO_2_ evolution, from 4.2 to 4.8 V. However, it should be noted that the ^18^O exchange treatment used by these authors results in partial ^18^O exchange both in the oxide and in the Li_2_CO_3_ impurity.^[68]^ Therefore, we favor the assignment for the low potential peak put forward by Yabuuchi et al. and by the Gasteiger group,[^66^, ^68^, ^73^, ^74^, ^75^, ^76^] who proposed that this peak results from a proton induced decomposition of Li_2_CO_3_, according to Equation 1.

(1) $$2\;H{}^{+}+\mathrm{Li}{}_{2}\mathrm{CO}{}_{3}\to \mathrm{CO}{}_{2}+H{}_{2}O+2\;\mathrm{Li}{}^{+}.$$

The protons are likely formed by oxidation of trace impurities in the electrolyte or of the electrolyte itself.^[66]^

The high‐potential CO_2_ peak, which appears at potentials above *E*≈4.6 V and essentially corresponds to the O_2_ evolution peak, is attributed to oxidation of the electrolyte by reaction with reactive (singlet) oxygen, which is released from the electrode upon activation of the Li_2_MnO_3_ domains.[^61^, ^66^, ^67^, ^68^, ^70^, ^72^, ^77^]

Plotting the MS signal against the potential highlights the strength of this DEMS setup. Here, the decrease of the signal at 4.5 V after the first maximum is clearly visible for W300 and A300, compared to the time dependent plot (Figure 7) and data given in the related, above‐discussed literature. Strehle et al. explained the decrease of the CO_2_ evolution rate with a limited quantity of possible reactants (impurities) for the reaction, being responsible for the first peak.^[68]^ However, from the location of the signal drop, it cannot be excluded that the decrease of the signal is only related to the constant potential at the flat plateau. Under the hypothesis that the CO_2_ evolving side reaction is related to the surface potential, the reaction has to reach a chemical equilibrium, which leads to a decrease of the gas evolution during the occurring phase transformation of the material at the plateau.

The remaining CO_2_ signal in the second cycle can be mainly attributed to different Li species, like Li_2_O_2_ or Li_2_CO_3_, which can be formed at the end of the discharge and to the reaction of further released oxygen at potentials >4.6 V with the electrolyte.^[73]^ This also means that in the second cycle sizable amounts of reactive oxygen are released, which, however, are fully consumed for reaction with the electrolyte. This is in accordance with the absence of the O_2_ signal. Consequently, the higher CO_2_ signal for A300 compared to W300 supports the observation of a faster capacity decrease of A300 due to a higher oxygen release.

For LRLO, the situation differs significantly from the W300 and A300 materials by the high amounts of Na_2_CO_3_ in the powder. There is the pronounced shoulder at ∼4.6–4.7 V, which is clearly visible in the plots vs. time (Figure 8a), but hardly resolved in the plot vs. potential (Figure 9d). This discrepancy is caused by the location of the shoulder in the range of the potential plateau region, with fast changes of the potential curve slope at the beginning and at the end of the plateau. However, the high CO_2_ evolution rate and the missing characteristic signal drop at 4.5 V in Figure 9d compared to A300 and W300 indicate that the CO_2_ evolution in LRLO is dominated by the Na_2_CO_3_ decomposition, analogous to Equation (1). A competing electrochemical oxidation of Na_2_CO_3_, which cannot be fully excluded from the present data, would not affect the signal shape in Figure 9d. Furthermore, the possible presence of a Na_2_CO_3_ cover layer can increase the reaction efficiency between the electrolyte and reactive oxygen species due to the slower off‐transport of the oxygen species and the related reaction products, like protons. This mechanism explains the much smaller O_2_ peak (first cycle) observed for LRLO compared to the treated W300 and A300 electrodes.

The CO_2_ signal of LRLO in the second cycle is lower compared to the first cycle, but still high relatively to the other electrodes. Most likely, the Na_2_CO_3_ impurities are not fully removed in the first cycle, further decomposition can occur in the second cycle and presumably also in the following ones. Additionally, it is possible that the amount of reactive oxygen evolved on the LRLO electrode in the second cycle is also significantly higher than on the other samples. This can be understood when considering that part of the surface was not accessible in the first cycle due to the Na_2_CO_3_ impurities, resulting in a successive activation of the material, which was already suggested during the electrochemical cycling.

Summarizing the DEMS results, the more pronounced O_2_ formation for the treated samples and the opposite trend for the CO_2_ formation clearly indicate that the Na_2_CO_3_ impurities were removed during the treatment and that the chemical activation of the material was successful. Additionally, the high CO_2_ formation of LRLO supports the assumption that during subsequent cycling the active material becomes more accessible and that the Na_2_CO_3_ impurities are not fully decomposed even after the first two cycles. Finally, the high O_2_ signal in the first and the increased CO_2_ signal in the second cycle of A300 underlines in combination with the decreasing discharge capacity during electrochemical cycling the hypothesis that the acidic treatment was too aggressive and therefore A300 undergoes a faster surface densification into a structure similar to A900.

## Conclusion

Spherical, Co‐free Li_1.2_Mn_0.6_Ni_0.2_O_2_ was synthesized using a scalable process. Post‐treatment of the as‐synthesized material with water or diluted HNO_3_, followed by calcination at 300 °C and 900 °C, respectively, had a strong effect on the electrochemical performance. Chemical impurities from the synthesis process were eliminated independently from the applied washing procedure. The effect of the post‐treatment steps could be classified into two groups. While the 300 °C treated samples were electrochemically activated (up to 270 mAh g^−1^), heat treatment at 900 °C lead to deactivation of the materials. The effect of the post‐treatment increased with higher acidity of the washing media, leaching more metal ions out of the materials, and thus leading to local Mn‐ and Ni‐enrichments. The activation could be explained by the formation of a thin spinel phase on top of the surface of the primary crystallites. This was indicated by the increasing fraction of Mn^III^ ions detected by XPS analysis, and supported by XRD and Raman spectroscopy. DEMS measurements showed an increased O_2_ release during the first delithiation with a simultaneously reduced CO_2_ formation, which underlined the complete removal of Na_2_CO_3_. It was clearly shown that the deactivation of the materials calcinated at 900 °C was caused by the phase separation of the powders into Mn‐enriched LRLO particles and Ni‐rich rock‐salt structures, such as Ni_6_MnO_8_. The latter was located predominantly at the grain boundaries and the surface regions of the crystallites and it was assumed to block Li^+^ diffusion pathways. Overall, this work showed that simple post‐treatment steps could have significant positive impact on the electrochemical performance of Co‐free LRLO material. Critical parameters, like the pH of the applied solution or the temperature of the subsequent heat treatment, have a strong influence on the electrochemical behavior and need to be adjusted carefully.

## Experimental Section

### Synthesis of the pristine material and post treatment steps

The pristine powder Li_1.2_Mn_0.6_Ni_0.2_O_2_ (LRLO) was synthesized by coprecipitation route. In a continuous stirred tank reactor (CSTR, *V*=1 L) a solution of manganese and nickel nitrate (Roth) was mixed under vigorous stirring with a NH_4_OH (Roth), a Na_2_CO_3_ (Roth) and a small amount of NaOH (Roth, to adjust the pH) solution. The excess suspension was filtrated continuously. The resulting spherical precursor material (Mn/Ni=3 : 1) was washed extensively with deionized water, dried and a small amount was mixed with stoichiometric amount of LiOH x H_2_O (Roth, Li/M=1.50). The final calcination step was conducted at 900 °C under air.

The different post treatment steps were done in a similar way, using ultrapure H_2_O (*σ* <0.55 μS cm^−1^). For the water treatment, pristine LRLO (5.5 g) was mixed with hot H_2_O (20 mL), stirred for 20 min and finally the water was decanted. This procedure was repeated three times and after the last residence time the suspension was filtrated and the powder was dried (120 °C, overnight). For acidic treatment HNO_3_ (65 %, Merck) was used. LRLO (5.5 g) was mixed with H_2_O (RT, 20 mL), acid was carefully added until the pH of the solution reached a value of pH=7, the suspension was stirred for 20 min and afterwards the solution was decanted, too. In the second and third repetition, only H_2_O without additional acid was used. As a last step, the mixture was filtrated and the solid residue was dried (120 °C, overnight). Both water and acid treated powders were divided in two equal portions. They were calcinated at 300 °C (W300 and A300) as well as at 900 °C (W900 and A900).

### Investigation of the post‐treatment procedure

Both types of washing solutions were collected, filtrated, acidified with HNO_3_ (conc., p.a.), diluted to a defined volume and analyzed by ICP‐OES (Spectro Arcos SOP). For further understanding of the heat treatment step after washing, the freshly washed powders were dried overnight under vacuum (80 °C) and subsequently TGA‐DSC‐MS measurements (heating rate: 10 K min^‐1^) were conducted in Al_2_O_3_ crucibles under air, using a TGA‐DSC device (Netzsch STA 449 C) coupled with a mass spectrometer (Netzsch QMS 403). DSC measurements were corrected with a blank measurement under the same conditions. The same powders were used without further heat treatment for the quantification of surface carbonates and hydroxides through acid‐base titration measurements. 0.20 g of powder was stirred in degassed ultrapure H_2_O (*σ* <0.55 μS cm^−1^, 30 mL, 7 min) under a N_2_ gas flow to avoid further carbonate contamination. The suspension was filtrated and the filtrate was instantaneously titrated with hydrochloric acid (0.01 m, OMNIS titrator, Metrohm) under N_2_ gas flow. The equivalence points (EPs) were determined using potentiometric endpoint detection with a pH electrode. The amount of carbonate and hydroxide ions were calculated from the EPs based on the method of Warder.^[78]^ The evolution of the pH after adding the LRLO powder (2.0 g) into water or acid containing solution (12 mL), respectively, was measured with a calibrated pH electrode and recorded by the OMNIS titration system at room temperature.

### Structural and chemical characterization

The chemical characterization of the powders was done by ICP‐OES measurements of a diluted aqua regia digestion (Spectro Arcos SOP) and cerimetric redox titration. For the latter, the amount of transition metal in the powders was determined through complexometric back titration of Na‐EDTA with a Cu^II^ solution, buffering the pH with NH_4_^+^/NH_3_ buffer. In a second titration step the powder was digested in an acidic (NH_4_)_2_Fe(SO_4_)_2_ solution under nitrogen and the remaining Fe^II^ was back titrated with Ce^IV^ solution. All titration experiments were conducted with an OMNIS titrator (Metrohm). The oxygen stoichiometry was calculated by the combination of the ICP and cerimetry results. XRD measurements were performed using a D8 Advance diffractometer (Bruker AXS, Bragg‐Brentano geometry in reflection mode, Cu‐K_α_ radiation, LYNXEYE XE detector). TOPAS V6 was used for Rietveld refinement of the diffraction data, using a Li_1.2_Mn_0.6_Ni_0.2_O_2_ structural model of Fell et al.^[41]^ For A900 an additional Ni_6_MnO_8_ structural model was applied.^[46]^ SEM images were obtained by using a Leo 1530 VP (Zeiss) scanning electron microscope with an acceleration voltage of 5 kV and an Everhart‐Thornley‐detector. The cross‐section of the particles was prepared by mixing the powder with an epoxy resin, applying the mixture on a support substrate and polishing the surface with a broad Ar^+^ ion beam (IM4000Plus, Hitachi). The Raman investigations of the powders and the cross section were carried out with an alpha300 R (WITec) confocal Raman microscope over a spectral range between −70 and 1108 rel. cm^−1^. The system consists of a spectrometer with a grating of 1800 l mm^−1^ and a CCD‐camera (1600×200 pixels) as well as a Nd:YAG laser with an excitation wavelength of 532 nm. The laser power was adjusted to 1.5 mW after microscope transit. The powders were measured using a 10x objective and for the cross sections a 100x objective was used (both Zeiss).

Electron transparent TEM samples were prepared using a focused Ga‐ion beam (FIB) system (Zeiss, type NVision 40). The surface of a typical sample particle of about 15 μm in diameter was protected using carbon deposition and applying the in situ gas injection (GIS) system integrated into the FIB. A slab of material of about 10 μm by 4 μm by 10 μm (length, width, depth) was etched out of the particle using a 30 kV Ga‐ion beam. This slab was lifted out and welded to a TEM lift out grid (Pelco company) using a piezo micromanipulator. The sample was subsequently thinned down to a final thickness of about 50 to 80 nm. The Ga‐ion beam current was reduced and the voltage was lowered to 5 kV to minimize amorphization damage.

The TEM investigations were carried out using a Talos 200X STEM (Thermofisher) operated at 200 kV. The system was equipped with an energy dispersive X‐ray (EDX) detector (type Thermofisher SuperX). TEM images were acquired using a CMOS camera (16 M pixels, type Thermofisher CETA). HAADF images together with EDX mappings were acquired to determine the local elemental composition. HRTEM images and diffraction pattern were used to determine the crystal structure.

For the XPS measurements, a commercial XPS machine from Physical Electronics (PHI 5800 ESCA) equipped with a hemispherical electron analyzer, a monochromatic Al K_α_ X‐ray source (1486.6 eV) and a flood gun to avoid charging of the sample was used. Survey spectra were recorded using a pass energy of 93.9 eV, detail spectra with 29.35 eV. Both angles (angle of photon incidence on the sample and angle of emitted photoelectrons) are 45° with respect to the surface normal (sample holder, respectively). The binding energies (BEs) of all spectra were calibrated with respect to the C 1s peak of ubiquitous carbon, which was fixed at a binding energy (BE) of 284.8 eV. The data were evaluated (deconvolution of spectra) by using the commercial software package CasaXPS (Casa Software Ltd., version 2.3.23PR1.0). The fitting of the Mn 2p spectra was performed according to data by Biesinger et al.^[79]^ for the Mn 2p, Mn 3s, and Mn 3p spectra, we additionally refer to the publication by Ilton et al.^[55]^

### Electrochemical characterization

A homogeneous slurry of each active material, polyvinylidene difluoride (PVDF, Solef 5130, Solvay) and conductive carbon Super C45 (Timcal) (dry ratio of 85 : 5 : 10) dispersed in an adequate amount of *N*‐methyl‐2‐pyrrolidinone (NMP, Sigma Aldrich) was coated on an aluminum foil using the doctor blade technique. After drying, disk electrodes with a diameter of 12 mm were punched out, pressed with a hydraulic press (8 t cm^‐2^ for 60 s), dried overnight under vacuum (120 °C) and transferred directly without contact to air into an argon‐filled glovebox (MBraun, O_2_ and H_2_O <0.1 ppm). Subsequently, the CR2032 coin cells (Hohsen) were assembled with one disk electrode, a lithium metal foil (450 μm, 12 mm diameter), two glass fiber coins (16 mm diameter, Whatman) as a separator and electrolyte (150 μL, 1 m LiPF_6_ in EC/DMC=1 : 1 by wt.). The typical active material loading of the electrodes was between 3 and 4 mg. The cells were cycled between 2.5 and 4.8 V using a BaSyTec galvanostat at room temperature. The applied currents are presented in the corresponding figures (1 C=250 mA g^−1^). The presented capacities of the electrochemical measurements are mean values of at least three cells.

The design of the DEMS cell used was similar to that already reported.^[65]^ Differently from the previous design, for contacting the working electrode an Al O‐ring and Al wire were used. The steel frit underneath the membrane was exchanged by perforated poly‐ether‐ether‐ketone (PEEK) plate to avoid side reactions. The electrode preparation was done similarly to the electrodes for electrochemical testing but on an Al coated (ALD) FEP membrane (50 μm thickness, Bohlender, Bola) instead of Al foil. The electrodes of 12 mm in diameter were punched from the membrane, dried at 100 °C under vacuum in a glovebox under argon atmosphere (LabMaster, MBraun, water and oxygen content <0.5 ppm) before use. Li metal stripes were used as counter and reference electrodes, respectively. They were cut from lithium foil (purity 99.9 %, Alfa Aesar) and separated in the cell by a Teflon plate. The bottom part was connected to the ultrahigh vacuum (UHV) chamber inlet of the mass spectrometer (Pfeiffer Vacuum QMA 410) to monitor the gas evolution upon cycling differentially without using any carrier gas. LP30 electrolyte (1 m LiPF_6_ in EC/DMC=1 : 1 by wt, Solvionic, purity >99.9 %, water content <20 ppm) was filled into the DEMS cell (0.7 mL). The assembly of the cell and all measurements were performed in an Ar filled glovebox (LabMaster Pro, MBraun, oxygen content <0.1 ppm, water content <0.5 ppm). Prior to the measurements the electrodes were resting in the electrolyte‐filled cell for at least two hours, to achieve stable OCP values (typically, around 3.0 V vs. Li^+^/Li) and stable background signals of the selected ion currents. A Princeton Research Instruments (PAR) 263 A potentiostat was used to conduct the galvanostatic experiments with a current of 0.1 mA, which corresponds to the C/10 rate at the applied loading of the active material, at cut‐off potentials 2.5 and 4.8 V (vs. Li^+^/Li), simultaneously measuring the intensity of selected ion currents.

## Conflict of interest

The authors declare no conflict of interest.

1

## Supporting information

As a service to our authors and readers, this journal provides supporting information supplied by the authors. Such materials are peer reviewed and may be re‐organized for online delivery, but are not copy‐edited or typeset. Technical support issues arising from supporting information (other than missing files) should be addressed to the authors.

Supporting Information

## Data Availability

The data that support the findings of this study are available from the corresponding author upon reasonable request.

## References

[1] M. Child , D. Bogdanov , C. Breyer , Energy Procedia 2018, 155, 44.

[2a] B. Nykvist , M. Nilsson , Nat. Clim. Change 2015, 5, 329;

[2b] M. Marinaro , D. Bresser , E. Beyer , P. Faguy , K. Hosoi , H. Li , J. Sakovica , K. Amine , M. Wohlfahrt-Mehrens , S. Passerini , J. Power Sources 2020, 459, 228073.

[3] A. Manthiram , J. C. Knight , S.-T. Myung , S.-M. Oh , Y.-K. Sun , Adv. Energy Mater. 2016, 6, 1501010.

[4] B. Qiu , M. Zhang , L. Wu , J. Wang , Y. Xia , D. Qian , H. Liu , S. Hy , Y. Chen , K. An , Y. Zhu , Z. Liu , Y. S. Meng , Nat. Commun. 2016, 7, 12108. 27363944 10.1038/ncomms12108 PMC4932185

[5] W. Zuo , M. Luo , X. Liu , J. Wu , H. Liu , J. Li , M. Winter , R. Fu , W. Yang , Y. Yang , Energy Environ. Sci. 2020, 13, 4450.

[6] R. A. House , G. J. Rees , M. A. Pérez-Osorio , J.-J. Marie , E. Boivin , A. W. Robertson , A. Nag , M. Garcia-Fernandez , K.-J. Zhou , P. G. Bruce , Nat. Energy 2020, 5, 777.

[7] M. M. Thackeray , C. S. Johnson , J. T. Vaughey , N. Li , S. A. Hackney , J. Mater. Chem. 2005, 15, 2257.

[8] Z. Lu , Z. Chen , J. R. Dahn , Chem. Mater. 2003, 15, 3214.

[9] H. Koga , L. Croguennec , P. Mannessiez , M. Ménétrier , F. Weill , L. Bourgeois , M. Duttine , E. Suard , C. Delmas , J. Phys. Chem. C 2012, 116, 13497.

[10a] W. Hua , S. Wang , M. Knapp , S. J. Leake , A. Senyshyn , C. Richter , M. Yavuz , J. R. Binder , C. P. Grey , H. Ehrenberg , S. Indris , B. Schwarz , Nat. Commun. 2019, 10, 5365; 31772159 10.1038/s41467-019-13240-z PMC6879514

[10b] F. Wu , G.-T. Kim , T. Diemant , M. Kuenzel , A. R. Schür , X. Gao , B. Qin , D. Alwast , Z. Jusys , R. J. Behm , D. Geiger , U. Kaiser , S. Passerini , Adv. Energy Mater. 2020, 10, 2001830;

[10c] Y. Li , M. J. Zuba , S. Bai , Z. W. Lebens-Higgins , B. Qiu , S. Park , Z. Liu , M. Zhang , L. F. Piper , Y. S. Meng , Energy Storage Mater. 2021, 35, 99.

[11a] P. K. Nayak , J. Grinblat , M. Levi , E. Levi , S. Kim , J. W. Choi , D. Aurbach , Adv. Energy Mater. 2016, 6, 1502398;

[11b] F. Wu , G.-T. Kim , M. Kuenzel , H. Zhang , J. Asenbauer , D. Geiger , U. Kaiser , S. Passerini , Adv. Energy Mater. 2019, 9, 1902445;

[11c] G. Chen , J. An , Y. Meng , C. Yuan , B. Matthews , F. Dou , L. Shi , Y. Zhou , P. Song , G. Wu , D. Zhang , Nano Energy 2019, 57, 157;

[11d] J. An , L. Shi , G. Chen , M. Li , H. Liu , S. Yuan , S. Chen , D. Zhang , J. Mater. Chem. A 2017, 5, 19738.

[12a] C. P. Laisa , R. N. Ramesha , K. Ramesha , Electrochim. Acta 2017, 256, 10;

[12b] X. Jin , Q. Xu , H. Liu , X. Yuan , Y. Xia , Electrochim. Acta 2014, 136, 19;

[12c] H. Xu , S. Deng , G. Chen , J. Mater. Chem. A 2014, 2, 15015.

[13] D. Chen , F. Zheng , L. Li , M. Chen , X. Zhong , W. Li , L. Lu , J. Power Sources 2017, 341, 147.

[14a] Y. Wu , J. Ming , L. Zhuo , Y. Yu , F. Zhao , Electrochim. Acta 2013, 113, 54;

[14b] M. Xu , Z. Chen , L. Li , H. Zhu , Q. Zhao , L. Xu , N. Peng , L. Gong , J. Power Sources 2015, 281, 444;

[14c] C. Zhou , P. Wang , B. Zhang , J. Zheng , Y. Zhou , C. Huang , X. Xi , J. Electrochem. Soc. 2018, 165, A1648-A1655.

[15] X. Zhang , R. Yu , Y. Huang , X. Wang , Y. Wang , B. Wu , Z. Liu , J. Chen , ACS Sustainable Chem. Eng. 2018, 6, 12969.

[16] Y. Lee , J. Lee , K. Y. Lee , J. Mun , J. K. Lee , W. Choi , J. Power Sources 2016, 315, 284.

[17] U. Breddemann , E. M. Erickson , V. Davis , F. Schipper , M. Ellwanger , M. Daub , A. Hoffmann , C. Erk , B. Markovsky , D. Aurbach , I. Krossing , ChemElectroChem 2019, 6, 3337.

[18] X. Ding , D. Luo , J. Cui , H. Xie , Q. Ren , Z. Lin , Angew. Chem. Int. Ed. 2020, 59, 7778. 10.1002/anie.202000628 32030866

[19] W. Guo , C. Zhang , Y. Zhang , L. Lin , W. He , Q. Xie , B. Sa , L. Wang , D.-L. Peng , Adv. Mater. 2021, 33, e2103173. 34337804 10.1002/adma.202103173

[20] S. Han , B. Qiu , Z. Wei , Y. Xia , Z. Liu , J. Power Sources 2014, 268, 683.

[21] H. Sclar , J. Sicklinger , E. M. Erickson , S. Maiti , J. Grinblat , M. Talianker , F. Amalraj Susai , L. Burstein , H. Beyer , L. Hartmann , G. Avruschenko , H. A. Gasteiger , B. Markovsky , D. Aurbach , J. Electrochem. Soc. 2020, 167, 110563.

[22a] J. Zhang , Z. Lei , J. Wang , Y. NuLi , J. Yang , ACS Appl. Mater. Interfaces 2015, 7, 15821; 26079270 10.1021/acsami.5b02937

[22b] P. Oh , S. Myeong , W. Cho , M.-J. Lee , M. Ko , H. Y. Jeong , J. Cho , Nano Lett. 2014, 14, 5965. 25180657 10.1021/nl502980k

[23] S.-H. Kang , C. S. Johnson , J. T. Vaughey , K. Amine , M. M. Thackeray , J. Electrochem. Soc. 2006, 153, A1186.

[24] S. Ramakrishnan , B. Park , J. Wu , W. Yang , B. D. McCloskey , J. Am. Chem. Soc. 2020, 142, 8522. 32271554 10.1021/jacs.0c02859

[25] D. Y. Yu , K. Yanagida , J. Electrochem. Soc. 2011, 158, A1015.

[26a] M. Metzger, H. Beyer, J. Sicklinger, D. Pritzl, B. Strehle, H. A. Gasteiger, H. Sclar, E. M. Erickson, F. Amalraj, J. Grinblat, D. Aurbach, WO2019/002116 A1, **2019**;

[26b] J. Sicklinger , H. Beyer , L. Hartmann , F. Riewald , C. Sedlmeier , H. A. Gasteiger , J. Electrochem. Soc. 2020, 167, 130507.

[27] K. Xu , S. Pang , Y. Wang , X. Shen , H. Wen , W. Wang , Y. Su , X. Xi , J. Electrochem. Soc. 2017, 164, A2348-A2354.

[28a] S. Pang , M. Zhu , K. Xu , X. Shen , H. Wen , Y. Su , G. Yang , X. Wu , S. Li , W. Wang , X. Xi , H. Wang , J. Electrochem. Soc. 2018, 165, A1897-A1902;

[28b] B. Liu , Int. J. Electrochem. Sci. 2018, 7578.

[29] M.-J. Wang , A.-F. Shao , F.-D. Yu , G. Sun , D.-M. Gu , Z.-B. Wang , ACS Sustainable Chem. Eng. 2019, 7, 12825.

[30] I. Hamam , N. Zhang , A. Liu , M. B. Johnson , J. R. Dahn , J. Electrochem. Soc. 2020, 167, 130521.

[31] M. Hartman , O. Trnka , V. Veselý , K. Svoboda , Chem. Eng. Commun. 2001, 185, 1.

[32] J.-W. Kim , H.-G. Lee , Metall. Mater. Trans. B 2001, 32, 17.

[33] I. A. Reyes , M. Flores , E. G. Palacios , H. Islas , J. C. Juárez , M. Reyes , A. M. Teja , C. A. Pérez , Minerals 2021, 11, 34.

[34] M. A. Rhamdhani , E. Jak , P. C. Hayes , Metall. Mater. Trans. B 2008, 39, 218.

[35] H. Henmi , M. Mori , T. Hirayama , N. Mizutani , M. Kato , Thermochim. Acta 1986, 104, 101.

[36a] J. Pan , Y. Sun , P. Wan , Z. Wang , X. Liu , Electrochem. Commun. 2005, 7, 857;

[36b] D. Pritzl , T. Teufl , A. T. S. Freiberg , B. Strehle , J. Sicklinger , H. Sommer , P. Hartmann , H. A. Gasteiger , J. Electrochem. Soc. 2019, 166, A4056-A4066.

[37a] P. Strobel , B. Lambert-Andron , J. Solid State Chem. 1988, 75, 90;

[37b] M. H. Rossouw , M. M. Thackeray , Mater. Res. Bull. 1991, 26, 463.

[38] T. Zhao , S. Chen , R. Chen , L. Li , X. Zhang , M. Xie , F. Wu , ACS Appl. Mater. Interfaces 2014, 6, 21711. 25402183 10.1021/am506934j

[39] A. Rougier , P. Gravereau , C. Delmas , J. Electrochem. Soc. 1996, 143, 1168.

[40a] Z. Yang , H. Zhou , Z. Bao , J. Li , C. Yin , J. Mater. Sci. Mater. Electron. 2019, 30, 19493;

[40b] K. Xu , S. Pang , Y. Wang , X. Shen , H. Wen , W. Wang , Y. Su , X. Xi , J. Electrochem. Soc. 2017, 164, A2348-A2354.

[41] C. R. Fell , D. Qian , K. J. Carroll , M. Chi , J. L. Jones , Y. S. Meng , Chem. Mater. 2013, 25, 1621.

[42] T. Nakamura , H. Gao , K. Ohta , Y. Kimura , Y. Tamenori , K. Nitta , T. Ina , M. Oishi , K. Amezawa , J. Mater. Chem. A 2019, 7, 5009.

[43] D. Marrocchelli , S. R. Bishop , H. L. Tuller , B. Yildiz , Adv. Funct. Mater. 2012, 22, 1958.

[44] W. Lee , D. Lee , Y. Kim , W. Choi , W.-S. Yoon , J. Mater. Chem. A 2020, 8, 10206.

[45] H. Taguchi , S. Omori , M. Nagao , H. Kido , M. Shimada , J. Solid State Chem. 1995, 118, 112.

[46] H. Taguchi , A. Ohta , M. Nagao , H. Kido , J. Solid State Chem. 1998, 135, 322.

[47] C. Julien , Solid State Ionics 2000, 136–137, 887.

[48] R. E. Ruther , A. F. Callender , H. Zhou , S. K. Martha , J. Nanda , J. Electrochem. Soc. 2014, 162, A98.

[49] K. Ben-Kamel , N. Amdouni , A. Mauger , C. M. Julien , J. Alloys Compd. 2012, 528, 91.

[50] C. M. Julien , M. Massot , Mater. Sci. Eng. B 2003, 100, 69.

[51] M. H. Brooker , J. B. Bates , J. Chem. Phys. 1971, 54, 4788.

[52] P. Lanz , C. Villevieille , P. Novák , Electrochim. Acta 2013, 109, 426.

[53] J. Zhang , R. Hu , P. Dai , Z. Bai , X. Yu , M. Wu , G. Li , J. Mater. Sci. Mater. Electron. 2018, 29, 7510.

[54] G. E. Lloyd , Mineral. Mag. 1987, 51, 3.

[55] E. S. Ilton , J. E. Post , P. J. Heaney , F. T. Ling , S. N. Kerisit , Appl. Surf. Sci. 2016, 366, 475.

[56a] R. T. Haasch , D. P. Abraham , Surf. Sci. Spectra 2019, 26, 14010;

[56b] M. Sathiya , K. Ramesha , G. Rousse , D. Foix , D. Gonbeau , A. S. Prakash , M. L. Doublet , K. Hemalatha , J.-M. Tarascon , Chem. Mater. 2013, 25, 1121. 10.1039/c3cc46842a 24165856

[57] M. C. Militello , S. W. Gaarenstroom , Surf. Sci. Spectra 2001, 8, 207.

[58] H. Liu , Y. Yang , J. Zhang , J. Power Sources 2007, 173, 556.

[59] J. Zheng , P. Xu , M. Gu , J. Xiao , N. D. Browning , P. Yan , C. Wang , J.-G. Zhang , Chem. Mater. 2015, 27, 1381.

[60] X. Zhang , M. Lengyel , R. L. Axelbaum , AIChE J. 2014, 60, 443.

[61] S. Shen , Y. Hong , F. Zhu , Z. Cao , Y. Li , F. Ke , J. Fan , L. Zhou , L. Wu , P. Dai , M. Cai , L. Huang , Z. Zhou , J. Li , Q. Wu , S. Sun , ACS Appl. Mater. Interfaces 2018, 10, 12666. 29569902 10.1021/acsami.8b00919

[62a] J. Zhang , R. Gao , L. Sun , Z. Li , H. Zhang , Z. Hu , X. Liu , Phys. Chem. Chem. Phys. 2016, 18, 25711; 27711565 10.1039/c6cp03683j

[62b] Y. Zheng , L. Chen , Y. Su , J. Tan , L. Bao , Y. Lu , J. Wang , R. Chen , S. Chen , F. Wu , J. Mater. Chem. A 2017, 5, 24292.

[63] G. Assat , D. Foix , C. Delacourt , A. Iadecola , R. Dedryvère , J.-M. Tarascon , Nat. Commun. 2017, 8, 2219. 29263321 10.1038/s41467-017-02291-9 PMC5738393

[64] S. Gao , Y. Zhang , H. Zhang , D. Song , X. Shi , L. Zhang , New J. Chem. 2017, 41, 10048.

[65] Z. Jusys , M. Binder , J. Schnaidt , R. J. Behm , Electrochim. Acta 2019, 314, 188.

[66] A. T. Freiberg , J. Sicklinger , S. Solchenbach , H. A. Gasteiger , Electrochim. Acta 2020, 346, 136271.

[67] T. Teufl , B. Strehle , P. Müller , H. A. Gasteiger , M. A. Mendez , J. Electrochem. Soc. 2018, 165, A2718.

[68] B. Strehle , K. Kleiner , R. Jung , F. Chesneau , M. Mendez , H. A. Gasteiger , M. Piana , J. Electrochem. Soc. 2017, 164, A400-A406.

[69] P. Linstrom , W. G. Mallard , “Mass Spectra” in NIST Chemistry WebBook; National Institute of Standards and Technology: Gaithersburg, MD 20899, 2010.

[70] D. Streich , A. Guéguen , M. Mendez , F. Chesneau , P. Novák , E. J. Berg , J. Electrochem. Soc. 2016, 163, A964-A970.

[71] N. Guerrini , L. Jin , J. G. Lozano , K. Luo , A. Sobkowiak , K. Tsuruta , F. Massel , L.-C. Duda , M. R. Roberts , P. G. Bruce , Chem. Mater. 2020, 32, 3733.

[72] K. Luo , M. R. Roberts , N. Guerrini , N. Tapia-Ruiz , R. Hao , F. Massel , D. M. Pickup , S. Ramos , Y.-S. Liu , J. Guo , A. V. Chadwick , L. C. Duda , P. G. Bruce , J. Am. Chem. Soc. 2016, 138, 11211. 27498756 10.1021/jacs.6b05111

[73] N. Yabuuchi , K. Yoshii , S.-T. Myung , I. Nakai , S. Komaba , J. Am. Chem. Soc. 2011, 133, 4404. 21375288 10.1021/ja108588y

[74] M. Metzger , B. Strehle , S. Solchenbach , H. A. Gasteiger , J. Electrochem. Soc. 2016, 163, A1219-A1225.

[75] S. Meini , N. Tsiouvaras , K. U. Schwenke , M. Piana , H. Beyer , L. Lange , H. A. Gasteiger , Phys. Chem. Chem. Phys. 2013, 15, 11478. 23748698 10.1039/c3cp51112j

[76] R. Jung , M. Metzger , F. Maglia , C. Stinner , H. A. Gasteiger , J. Electrochem. Soc. 2017, 164, A1361-A1377.

[77a] A. T. S. Freiberg , M. K. Roos , J. Wandt , R. de Vivie-Riedle , H. A. Gasteiger , J. Phys. Chem. A 2018, 122, 8828; 30354136 10.1021/acs.jpca.8b08079

[77b] N. Mahne , S. E. Renfrew , B. D. McCloskey , S. A. Freunberger , Angew. Chem. Int. Ed. 2018, 57, 5529. 10.1002/anie.201802277 PMC5947587 29543372

[78a] A. A. Benedetti-Pichler , M. Cefola , Ind. Eng. Chem. Anal. Ed. 1939, 11, 327;

[78b] R. B. Warder , Chem. News 1881, 43, 228.

[79] M. C. Biesinger , B. P. Payne , A. P. Grosvenor , L. W. Lau , A. R. Gerson , R. S. Smart , Appl. Surf. Sci. 2011, 257, 2717.

