# Supplementary material for: Enhanced Electrochemical Capacity of Spherical Co‐Free Li1.2Mn0.6Ni0.2O2 Particles after a Water and Acid Treatment and its Influence on the Initial Gas Evolution Behavior
Source: ChemSusChem. 2022 Sep 8;15(20):e202201061. doi: 10.1002/cssc.202201061 (PMC9826533; doi:10.1002/cssc.202201061)
Supplement: Supplementary file 1 — Supporting Information [file CSSC-15-0-s001.pdf]

ChemSusChem

Supporting Information

Enhanced Electrochemical Capacity of Spherical Co-Free $Li_{1.2}Mn_{0.6}Ni_{0.2}O_2$ Particles after a Water and Acid Treatment and its Influence on the Initial Gas Evolution Behavior

Florian Klein, Joachim Bansmann, Zenonas Jusys, Claudia Pfeifer, Philipp Scheitenberger, Manuel Mundszinger, Dorin Geiger, Johannes Biskupek, Ute Kaiser, R. Jürgen Behm, Mika Lindén, Margret Wohlfahrt-Mehrens, and Peter Axmann*© 2022 The Authors. ChemSusChem published by Wiley-VCH GmbH. This is an open access article under the terms of the Creative Commons Attribution License, which permits use, distribution and reproduction in any medium, provided the original work is properly cited.

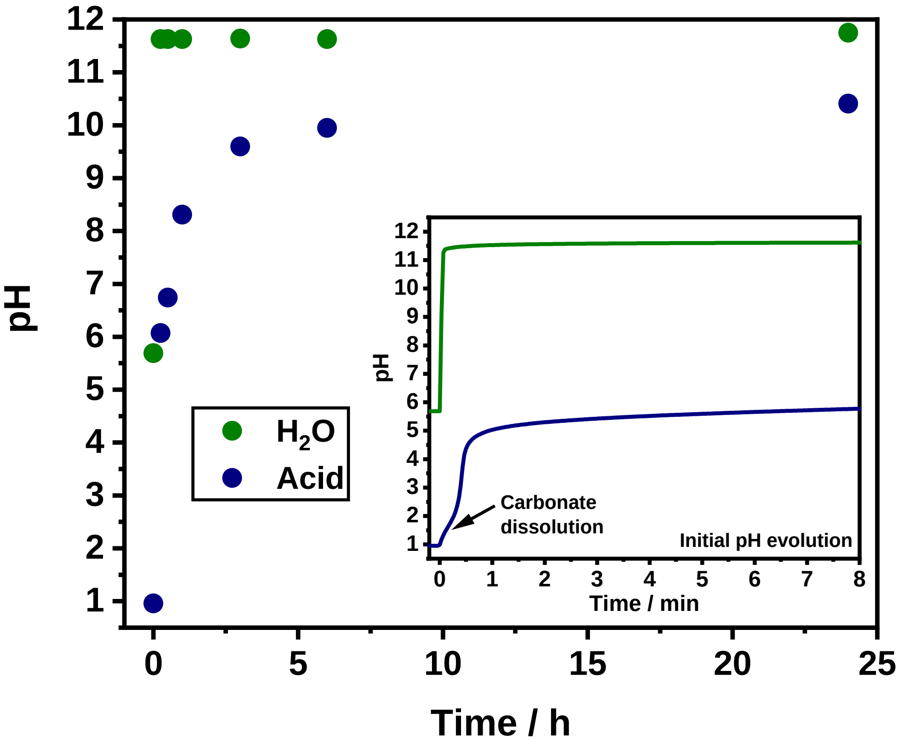

Figure S1. Evolution of the pH after adding LRLO powder into water (green) and into acidic solution (blue). Detailed initial evolution is presented in the inset.

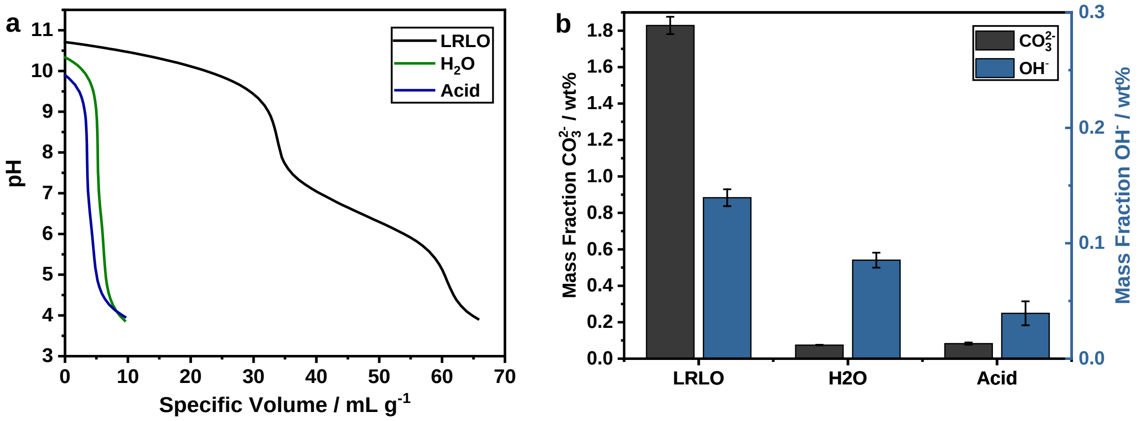

Figure S2. Investigation of the soluble surface species on the LRLO powder (black) after water (green) and acidic (blue) treatment without heat treatment: a) acid-base-titration curve with 0.01 M hydrochloric acid and b) quantitative analysis of carbonate (dark grey) and hydroxide ions (blue).

Table S1. Detailed results of the Rietveld refinement based on the structural model for $Li_{1.2}Mn_{0.6}Ni_{0.2}O_2$ of Fell et al.[1]: space group, lattice parameters, Cell Volume *V*, cation occupancies, atom coordinate *z* of the oxide ions, the estimated crystallite size (integral breadth) and the *R* factor.

	LRLO	W300	W900	A300	A900*
**Spacegroup**	*R-3m* (166)	*R-3m* (166)	*R-3m* (166)	*R-3m* (166)	*R-3m* (166)
a **/ Å**	2.858	2.859	2.860	2.863	2.849
c **/ Å**	14.257	14.258	14.262	14.274	14.264
**V / Å³**	100.9	100.9	101.1	101.3	100.2
**Li_{Li} (3a: 0, 0, 0)**	0.98	0.99	0.99	0.99	0.99
**Ni_{Li} (3a: 0, 0, 0)**	0.02	0.01	0.01	0.01	0.01
**Li_{TM} (3b: 0, 0, 1/2)**	0.23	0.22	0.18	0.18	0.18
**Mn_{TM} (3b: 0, 0, 1/2)**	0.59	0.60	0.60	0.63	0.64
**Ni_{TM} (3b: 0, 0, 1/2)**	0.18	0.18	0.18	0.19	0.18
z **[O (6c: 0, 0, z)]**	0.240	0.240	0.240	0.240	0.240
**Crystallite Size / nm**	108	120	146	93	181
**R_{wp} / %**	9.6	10.0	9.6	8.4	11.7

* additional Ni_6MnO_8 phase was used for the refinement based on Taguchi et al.[2]

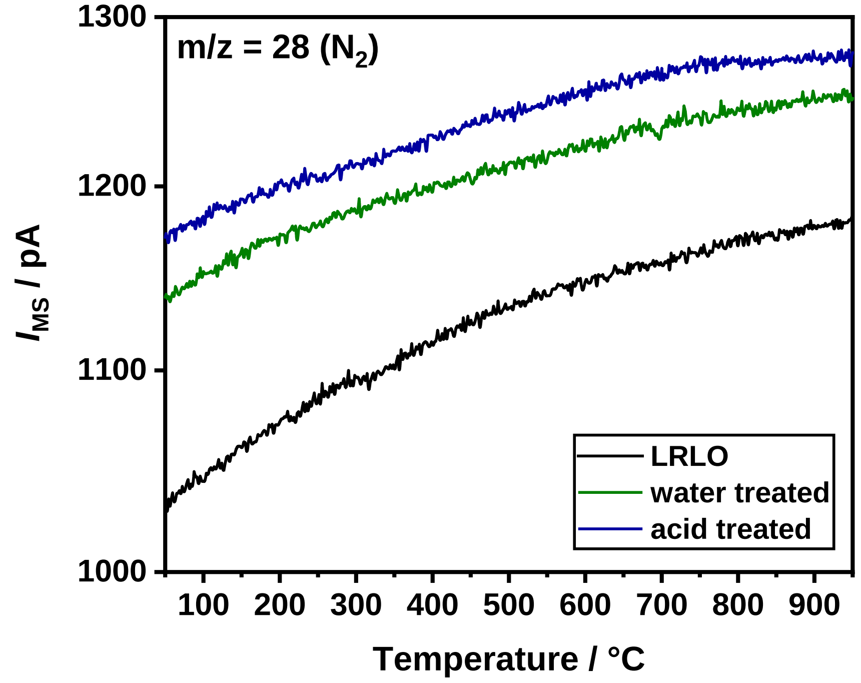

Figure S3. Corresponding MS curves to the TGA-DSC-MS experiments for m/z = 28 (N_2) measured with a heating rate of 10 K min^{-1} under air atmosphere for the as-synthesized LRLO powder (black), LRLO after aqueous washing (green) and LRLO after acidic treatment (blue).

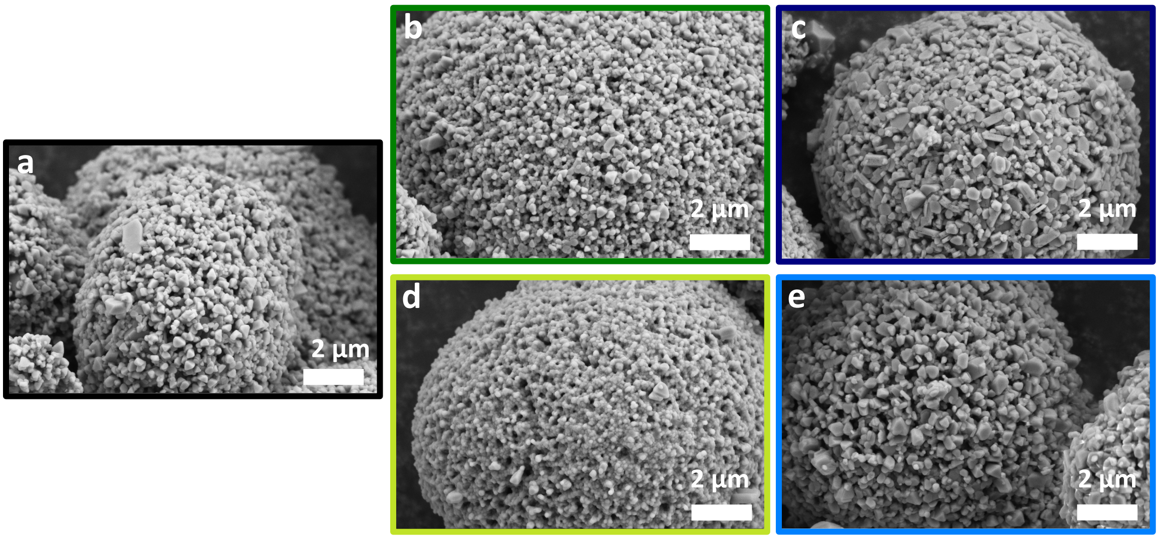

Figure S4. SEM images of the different synthesized materials: a) as-synthesized LRLO, b) W300, c) A300, d) W900 and e) A900.

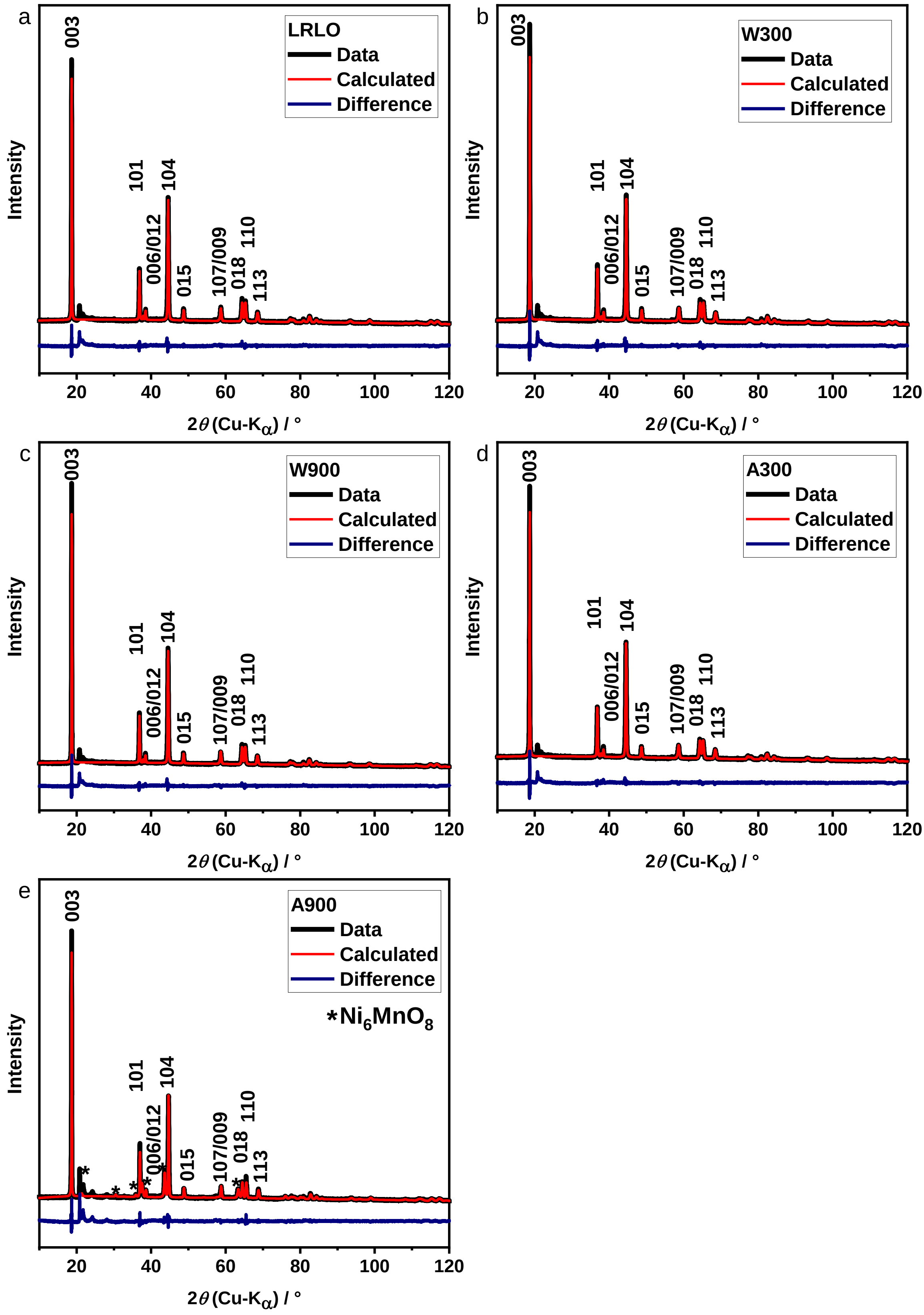

Figure S5. Comparison of the measured XRD pattern (black line) with the calculated pattern, using Rietveld refinement (red line) and their difference (blue line) of a) LRLO, b) W300, c) W900, d) A300 and e) A900.

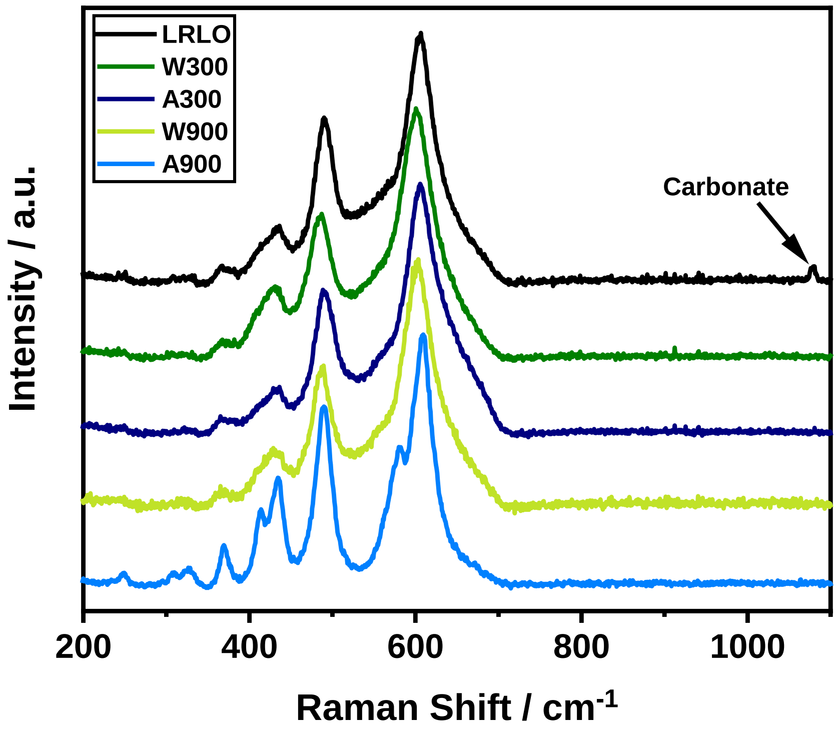

Figure S6. Raman overview spectra showing the non-existence of carbonates after the different treatments.

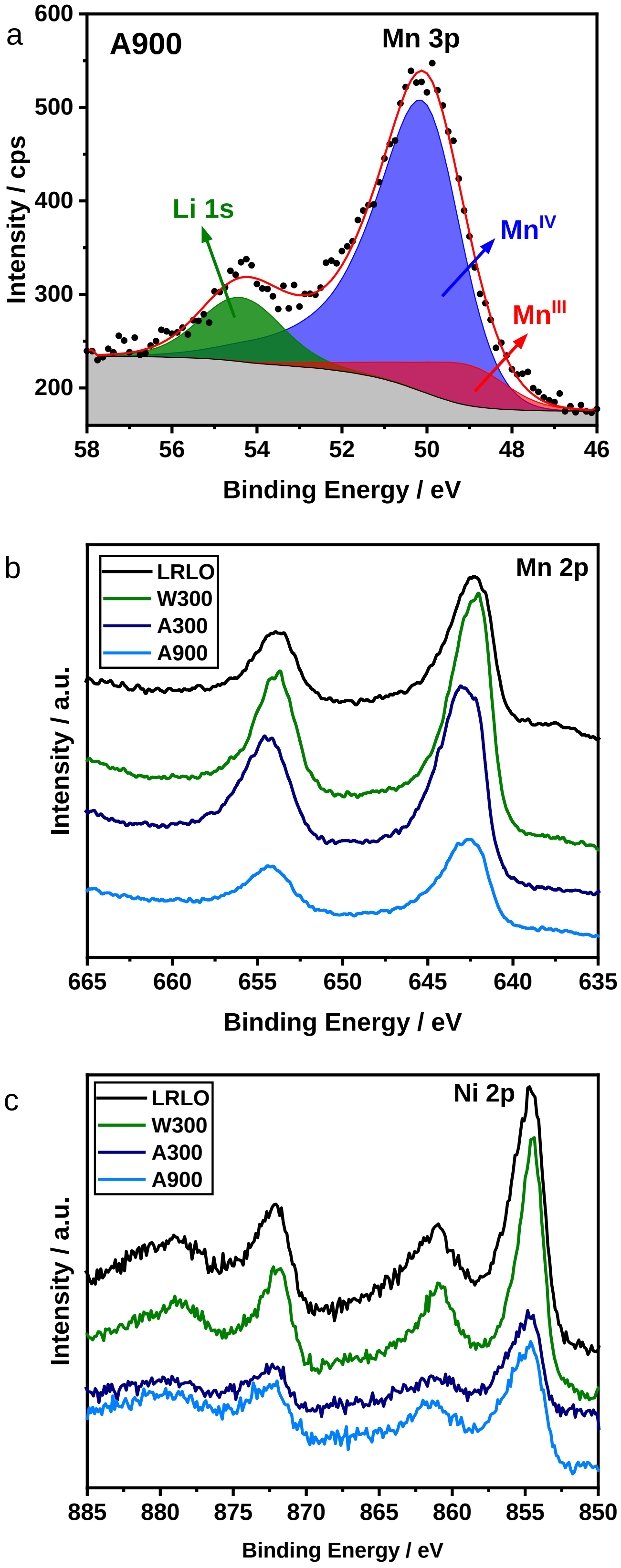

Figure S7. XPS spectra of a) A900 (Mn 3p and Li 1s) as well as LRLO, W300, A300 and A900: b) Mn 2p and c) Ni 2p

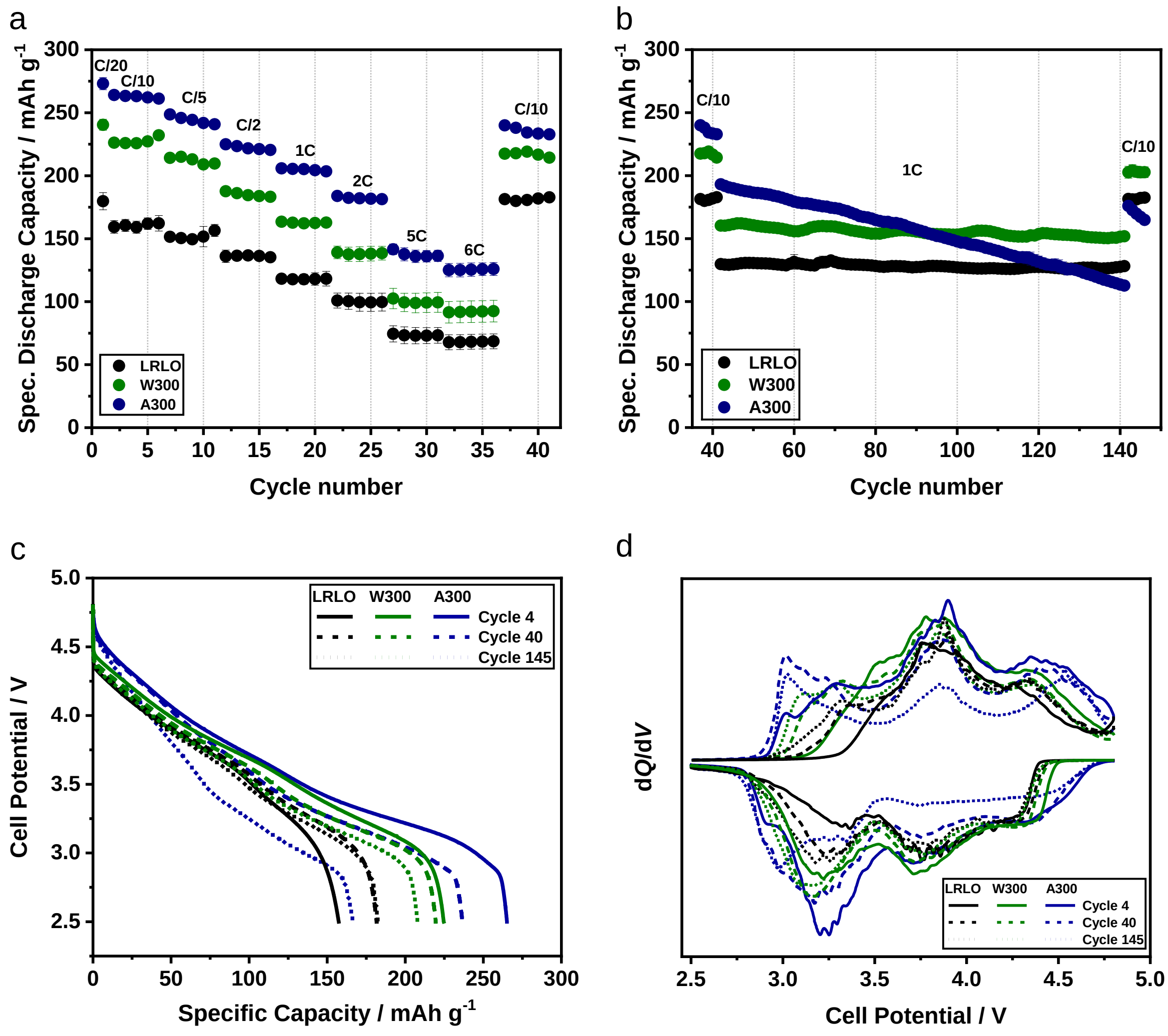

Figure S8. a) Rate capability test of LRLO, W300 and A300 with b) further cycling at 1C; c) Potential curves of LRLO, W300 and A300 of selected C/10 cycles during the long-term cycling and d) corresponding d*Q*/d*V* plots.

References

[1] C. R. Fell, D. Qian, K. J. Carroll, M. Chi, J. L. Jones, Y. S. Meng, *Chem. Mater.* **2013**, *25*, 1621.

[2] H. Taguchi, A. Ohta, M. Nagao, H. Kido, *J. Solid State Chem.* **1998**, *135*, 322.
